# Strengths, challenges, and variations - insights into biosecurity practices in Swedish poultry production following HPAI outbreaks

**DOI:** 10.1016/j.psj.2025.105871

**Published:** 2025-09-19

**Authors:** Malin Grant, Désirée S. Jansson, Arianna Comin, Magdalena Jacobson, Maria Nöremark

**Affiliations:** a Swedish Veterinary Agency, Department of epidemiology, disease surveillance and risk assessment, SE751 89, Uppsala, Sweden; b Swedish University of Agricultural Sciences, Department of Clinical Sciences, Section for pig and poultry medicine, Box 7054, SE750 07, Uppsala, Sweden

**Keywords:** Poultry, Biosecurity, Highly pathogenic avian influenza, Thematic analysis, Implementation

## Abstract

This study investigated quantitatively and qualitatively the implementation of biosecurity in commercial poultry production in Sweden during 2020 and 2021 when outbreaks of highly pathogenic avian influenza (HPAI) occurred. The study included case and non-case farms located in areas subjected to HPAI restriction zones with broiler parent breeders, layer pullets, laying hens, broilers, and meat turkeys with at least 2,000 birds. General biosecurity routines were investigated focusing on the wild bird-poultry interface. Data collection was based on face-to-face interviews and on-farm observations on 15 farms with HPAI outbreaks and 33 matched non-case farms using a questionnaire and the biosecurity scoring tool Biocheck.UGent (https://biocheckgent.com) to assess general biosecurity practices. Data were analyzed to identify differences related to poultry categories, geographical region, farm size and HPAI disease status. Additionally, qualitative data were examined using thematic analysis to explore barriers to biosecurity implementation.

The findings indicated that while biosecurity levels were generally high, there was significant variation among farms with category-specific strengths and challenges. Common weaknesses observed included inadequate infrastructure such as anteroom layout, limited training of farmworkers, suboptimal hand hygiene, and difficulties in maintaining good hygiene during the storage and introduction of roughage, such as hay and straw, into barns. Moreover, farmyards often lacked designated clean and dirty areas.

The qualitative analysis identified several factors affecting the implementation of biosecurity, and key qualitative themes were conflicting priorities, compliance based on perceived risk, and feelings of powerlessness. A need for specific knowledge on effective biosecurity measures against HPAI was expressed as well as lack of knowledge among farmworkers. The farm infrastructure could both facilitate and hamper effective biosecurity depending on its design. A risk-based approach meant adapting biosecurity based on the perceived risk of outbreaks and risk connected to different introduction routes. The conflicts of interest raised were often in relation to animal welfare and environmental considerations.

The main conclusions were that there is high heterogeneity in biosecurity among Swedish poultry farms, with implementation affected by multiple factors.

## Introduction

Biosecurity measures within the poultry industry not only mitigate the risk of infectious poultry diseases, foodborne zoonoses, and antimicrobial resistance, but also contribute to the sustainability of poultry production and enhanced animal welfare. Among the many reasons to maintain good biosecurity, the global spread of highly pathogenic avian influenza virus (HPAIV) in the recent decade has highlighted a need for improved biosecurity standards worldwide. Following the emergence of the A/Goose/Guangdong/1/1996 (Gs/Gd) H5N1 virus lineage, multiple events of intercontinental viral transmission along wild waterfowl migratory flyways (Lycett et al., 2019) have put biosecurity to the test also in Sweden. Since 2016, there have been 27 outbreaks of HPAI in Swedish poultry (European Commission, 2025), with the worst epidemic season in 2020/2021 (Grant et al., 2022). Virus were predominantly introduced through indirect contact with wild birds (Grant et al., 2022), a route of transmission that has been demonstrated by others (Bouwstra et al., 2015; Beerens et al., 2019; King et al., 2022; Nagy et al., 2022; Dziadek et al., 2024). The transmission mechanism is not fully understood, but the importance of different avian and mammalian bridging species has been highlighted (Root and Shriner, 2020; Shriner and Root, 2020). Although the risk of avian influenza virus (AIV) introduction from wild birds is higher in poultry with outdoor access (Gonzales et al., 2017), outbreaks also occur in poultry housed indoors on allegedly high biosecurity farms, demonstrating the importance of indirect transmission pathways via fomites, vectors and people. Airborne viral transmission between farms has been demonstrated (Ypma et al., 2013; Torremorell et al., 2016), whereas others assessed this way of transmission from wild birds to poultry to be less likely (de Vos and Elbers, 2024). Even with the launch of vaccination programs in some EU member states, biosecurity remains a cornerstone (EFSA AHAW Panel (EFSA Panel on Animal Health and Animal Welfare) et al., 2023a) and recommendations to prevent both HPAI and low pathogenic avian influenza (LPAI) entry and spread are available (EFSA AHAW Panel (EFSA Panel on Animal Health and Welfare) More et al., 2017).

Theoretical frameworks from human behavioral science have been adopted in the veterinary field to better understand factors influencing farmers' decision-making regarding animal disease control, and barriers to good practices (Ellis-Iversen et al., 2010; Garforth, 2015; Mankad, 2016; Renault et al., 2021). Socio-psychological determinants such as knowledge, attitudes and personality traits can be barriers for the implementation of biosecurity measures (Racicot et al., 2012; Delpont et al., 2021). Economic considerations, including costs and limited evidence of financial benefits, have also been identified as important barriers (Laanen et al., 2014; Rajala et al., 2024). Moreover, Pao et al. (2022) concluded that good biosecurity cannot be upheld only by efforts at farm level but requires coordinated support from other industry stakeholders and policymakers. While studies have examined biosecurity in other livestock species in Sweden (Nöremark et al., 2010; Nöremark and Lewerin, 2014; Backhans et al., 2015; Nöremark et al., 2016; Gröndal et al., 2023), research on biosecurity in Swedish poultry production is limited and has mainly focused on *Campylobacter* in broilers (Hansson et al., 2010).

**Fig. 1:**
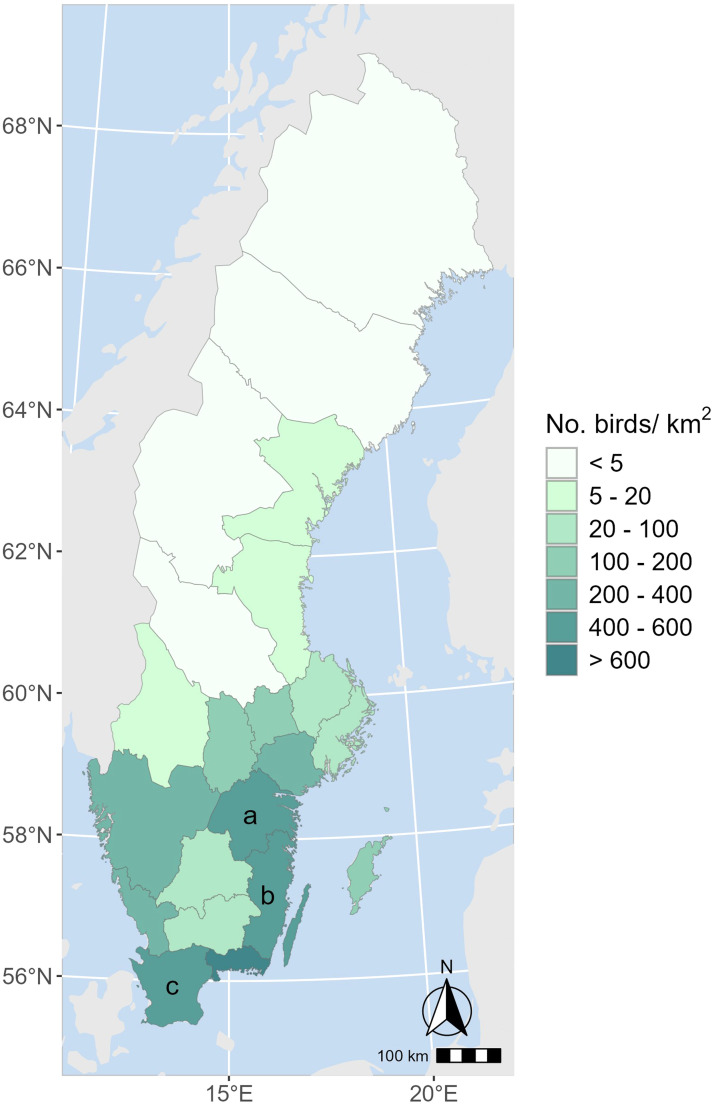
Map of Sweden showing the number of poultry per km^2^ and per county (the Swedish poultry register, extracted 26-03-2025, the Swedish Board of Agriculture). The farms in the study were located in the counties of Östergötland (a), Kalmar (b), and Skåne (c).

Sweden's poultry industry, concentrated in the south, is dominated by broiler and table egg production and is less densely populated compared to much of continental Europe (Fig. 1), including 8,0 million laying hens and 9,3 million broilers (Jordbruksverket, 2024c), with an annual production of 106,3 million broilers, 520,000 meat turkeys (Jordbruksverket, 2025) and 114,700 tonnes of eggs (Jordbruksverket, 2024a). Fourteen percent of table eggs and less than 1 % of poultry meat are produced on organic farms (Jordbruksverket, 2024b). Farms are mainly operated by individual companies that may manage more than one farm, often owning both the farm and the land. The majority of large-scale poultry operations are enrolled in biosecurity programs, with annual audits, managed by one of the poultry farmers’ organizations. The prevalence of *Salmonella* and *Campylobacter* spp. EFSA and ECDC (European Food Safety Authority and European Centre for Disease Prevention and Control) (2024) as well as antibiotic usage is low (European Medicines Agency, 2023). Furthermore, Sweden has hitherto been a non-vaccinating country as regards Newcastle disease, even if sporadic outbreaks have occurred.

The aims of this study were to investigate biosecurity practices and factors influencing their implementation on commercial poultry farms in Sweden. Insights will be used to improve infectious disease prevention. A mixed-method design was used as qualitative data is essential to capture the complex socio-psychological and structural drivers that quantitative indices alone cannot explain.

## Methods

### Recruitment of farms

Farms were eligible for inclusion if they had at least one flock diagnosed with HPAI (case farms) or were located within a 10-km radius restriction zone from an HPAI outbreak (non-case farms) during the period November 2020 to December 2021. Additionally, a farm should keep a minimum of 2,000 birds of either parent breeders, layer pullets, laying hens, broilers, or meat turkeys according to data from poultry industry organizations and the national poultry register. Eighteen case farms and 58 non-case farms located in three different geographical regions (Fig. 1) fulfilled the inclusion criteria. The farms were recruited in parallel for a separate study investigating risk factors for introduction of HPAIV to commercial poultry farms (Grant et al. in preparation).

As all farms were to be visited by the same person, travel logistics, biosecurity programs limiting farm visits to one per day, and project funding, set an upper limit for the total number of farms. It was decided to include all case farms and two non-case farms per case farm, matched by poultry category. All case farms were contacted, and non-case farms were listed and contacted in random order. Since there were too few non-case turkey farms, extra broiler farms were selected instead. One company operated 13 eligible farms, and only seven of these were selected to avoid over-representation. Farmers were invited by post, followed by telephone calls, text messages, and/or email if necessary.

### Questionnaire and Biocheck scoring tool

Data collection was based on a questionnaire focusing on HPAI biosecurity aspects, and the biosecurity scoring tool Biocheck UGent™ (Biocheck UGent™, 2021), hereafter named Biocheck. The questionnaire (Supplementary material 1) was developed based on previous epidemiological investigations in HPAI-outbreak flocks (Grant et al., 2022) and potential biosecurity risk factors described in the literature (Gonzales et al., 2017; Wells et al., 2017; Guinat et al., 2020). It also included questions about perceptions on HPAIV transmission and measures introduced to improve on-farm biosecurity following the outbreaks. The questionnaire was piloted on two animal health experts at The Swedish Veterinary Agency, one representative from the Swedish Egg Association, and one poultry farmer.

**Fig. 2:**
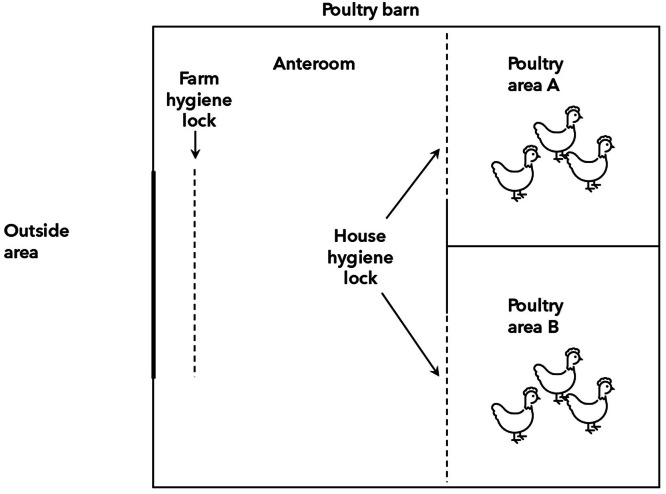
Definition of farm hygiene lock and house hygiene lock in this study. When only one hygiene lock per barn was available it was categorized as farm hygiene lock. Farms with multiple barns could have multiple farm hygiene locks according to the definition used.

Biocheck uses category-specific protocols, i.e. questions are adapted to the type of production, such as broilers or laying hens. Data are entered in an online tool and numerical scores are generated which can be used for intra- and inter-farm comparison of total, internal and external biosecurity, and biosecurity subcategories (Gelaude et al., 2014). At the time of this study, no protocol was available for meat turkey farms, and instead the protocol for broilers was used (Supplementary material 2). Similarly, the protocol for laying hens was used for both laying hens, broiler parent breeder farms and layer pullet farms (Supplementary material 3). Both Biocheck protocols were accessed in December 2021 and translated into Swedish. Relevant questions available in only one Biocheck protocol were also asked when using the other protocol. Moreover, some questions in Biocheck were expanded, e.g. in the question “washing and disinfecting hands” data was also collected separately for “washing hands” and “disinfecting hands”. The terms farm and house hygiene lock were defined according to Fig. 2 in this study. In Biocheck, farms are rewarded for a clean area delimited by a virtual or physical fence with controlled access through a farm hygiene lock, a setup that is rare in Sweden. Therefore, the definitions were adapted to distinguish between farms with one or two (or more) hygiene locks.

### Data collection

All farms were visited by the first author between May and November 2022. Each visit included an interview and observations, using the questionnaire and Biocheck. The interviewees were asked to report the circumstances that existed at the time of restriction zones for HPAI in 2020-2021, and any changes that had been made thereafter. The on-farm observations focused on the design and condition of the farmyard, roof, walls and doors of the poultry barns, feed silos, manure storage, carcass storage, ventilation inlets and outlets, and anterooms including hygiene locks. Anteroom layout was assessed based on 1) the direction of movement through the changing room and shower (if present), 2) ease of access to and location of handwashing facilities in relation to clean and dirty areas, 3) space allowance for changing clothes and boots, 4) design of the hygiene barrier and 5) clarity in the separation of clean and dirty areas.

The interviewee was either the farmer or farmworker most acquainted with daily farm operations and on some farms more than one person participated. If the interviewee lacked proficiency in Swedish or English, an additional employee assisted with translation. Qualitative information and comments beyond the questions were recorded as free text. In some cases, additional information and clarifications were obtained by email afterwards. All responses, observations and comments were documented on paper and later entered into a Microsoft Access database (2411 version) and the Biocheck online scoring tool, respectively.

### Statistical analyses

Farms were grouped by size based on the maximum bird capacity (data from the Swedish poultry register) as: small (0–33rd percentile), medium (33rd–66th percentile), and large (66th–100th percentile) per poultry category, Table 1. All data were processed in the statistical software environment R, version 4.4.1 (R Core Team, 2024). Individual variables were compared between poultry categories using Fisher’s exact test with a 5 % significance level. A simple linear regression model was used to compare the number of birds (natural log) to the total Biocheck score. Total score was compared between poultry categories (broilers vs fattening turkeys and laying hens vs broiler parent breeders), geographical regions, farm size, organic or conventional production, and sex of interviewee, using a t-test or one-way ANOVA with a 5 % significance level. Scores per biosecurity subcategory were compared pairwise between poultry categories as above, using a t-test. Total scores, external scores and individual variables representing the circumstances at the time of restriction zones, were compared between case farms and non-case farms using a t-test. In addition, total scores at the time of restriction zones, and after implementing changes, were compared using a t-test. For farms with layer pullets and broiler parent rearing stock, no Biocheck scores were obtained as multiple questions from the laying hen protocol didn’t apply.

**Table 1:** Farm size classification based on the maximum bird capacity.

Poultry category	Small	Medium	Large
Broilers	<87,000	87,000–140,000	>140,000
Laying hens/ Broiler parent breeder/ Layer pullets	<16,000	16,000–30,000	>30,000
Meat turkeys	<5,000	5,000–18,000	>18,000

### Qualitative analysis

All free-text comments, either related to a specific question or not, were used for a thematic analysis (Saunders et al., 2023). The comments were coded by the first author and organized into preliminary themes. All comments, codes, and preliminary themes were examined by the last author, discussed and revisited iteratively, resulting in final themes.

## Results

### Participating farms

In total, 66 farms were contacted of which 48 farms participated (Table 2). The overall participation rate was 73 % (88 % among HPAI case farms and 67 % among non-case farms). The most common reasons not to participate were time constraints and unwillingness to receive non-essential visitors. Thirteen farms (27 %) were organic laying hen or broiler farms. The farms were located in the counties of Kalmar (6 %), Skåne (63 %) and Östergötland (31 %) (Fig. 1). Parent breeder farms from both broiler and egg-producing sectors were invited, but only broiler parent breeder farms chose to participate. In the statistical analysis, the broiler parent farm category included one farm which raised parent chickens.

**Table 2:** Number of participating farms by poultry category and HPAI disease status in 2020 –2021, and numerical characteristics of participating farms by poultry category.

Poultry category	No. of farms^1^	No. of case farms^1^	No. of non-case farms^1^	Number of birds on the farm^2^	Years experience of keeping poultry^2^	Number of people working on the farm^2^	Age of the oldest poultry building in use (years)^2^	Age of the newest building in use (years)^2^
Broiler parent breeder	9	3	6	26,600 (12,000–85,000)	11 (4–47)	4 (2–30)	50 (27–60)	40 (8–50)
Layer pullets	2	1	1	417,500 (100,000–735,000)	30 (10–50)	3 (1–5)	32 (13–50)	27 (4–50)
Laying hens	15	5	10	27,000 (13,500–1,240,000)	14 (3–50)	3 (1–14)	14 (4–200)	12 (4–30)
Broilers	10	1	9	133,000 (19,200–250,000)	15 (5–31)	2 (1–5)	13 (5–100)	7 (1–22)
Meat turkeys	12	5	7	6,450 (2,350–35,000)	26 (13–33)	3 (2–7)	30 (12–100)	25 (2–100)
Total	48	15	33	24,200 (2,350–1,240,000)	20 (3–50)	3 (1–30)	26 (4–200)	13 (1–100)

1 Count.

2 Median (min–max).

The interviewees were farmers (*n* = 32 farms), farmworkers (*n* = 12 farms), or both farmers and farmworkers jointly (*n* = 4 farms). On 32 out of 48 farms the person(s) interviewed was a man, on eight farms a woman and on the remaining farms individuals of both sexes were interviewed together. The farms represented 33 individual poultry companies. On 34 farms, the interviewee(s) responded to the questions for a single farm. Five interviewees responded to questions for two farms each, and one interviewee provided answers for four different farms. Each farm was visited and observed independently.

### Biosecurity programs, plans and training

Of the visited farms, 43 (90 %) were affiliated to an industry biosecurity program managed by the Swedish Egg Association (35 %) or the Swedish Poultry Meat Association (55 %). Four farms (8 %) had a farm-specific, written biosecurity plan. The most frequently cited source of biosecurity advice was veterinarians (56 %), followed by advisors from poultry farmers' organizations (35 %). Farmers/farmworkers had participated in biosecurity training in the recent five years on 24 farms (50 %), with all farmworkers trained on seven farms and only the farmer trained on 17 farms. On 22 farms (46 %), two or more languages were spoken at work and on 14 farms (29 %) challenges due to language barriers were reported. On 24 farms (50 %), some farmworkers had a different mother tongue than Swedish, and on 12 of these, instructions were provided in their mother tongue. On 23 farms (48 %), biosecurity instructions in Swedish or another language were displayed in the buildings to guide farmworkers and visitors.

### Biosecurity measures

**Fig. 3:**
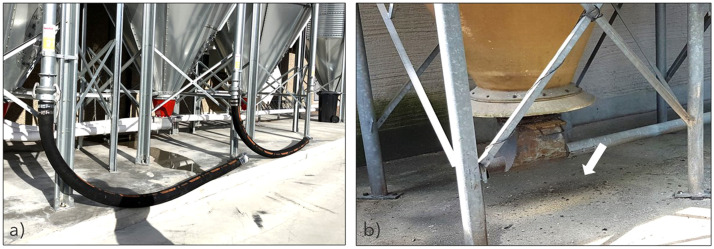
Different levels of hygiene below feed silos observed during farm visits: a. Very clean, b. Presence of feed spillage and bird droppings.

Implementation of biosecurity measures from the questionnaire and Biocheck are available in Table 3 (overall) and Table 4 (by poultry category). From a biosecurity perspective, 46 % of farms were assessed to have an optimal anteroom layout (Table 3). A difference was observed between broiler parent breeder farms and laying hen farms, where the former had stricter biosecurity at the farm hygiene lock (i.e. hygiene lock between outside areas and anteroom, Fig. 2), and laying hen farms had more emphasis on the house hygiene lock (i.e. hygiene lock between anteroom and poultry areas, Fig. 2; Table 4). A visitor’s log was available on 46 % of farms at the time of the visit, less commonly on broiler and meat turkey farms compared to the other poultry categories (Table 4). Boot or vehicle disinfection baths were not used, except on one farm (Table 3). On 29 % of farms, the catching team always used disposable or farm-specific shoes and clothing and this proportion was lower for broiler parent breeder farms and broiler farms compared to the other poultry categories (Table 4). Carcass collection was usually arranged without transport vehicles entering the farm, i.e. collection was made near a public road (Table 3). Half of the farms stored manure on-site, more commonly so on the laying hen farms (Table 4). Some broiler farms reported sharing equipment with other farms (Table 4). This was related to the use of the same catching machine to collect chickens for slaughter. Roughage (e.g. straw, hay, silage or lucerne) was supplied to birds on 46 % of farms (Table 3), mainly on organic and male meat turkey farms. Biosecurity routines for storage and delivery of roughage to barns varied (Table 3). Some poultry categories, in particular broiler parent breeder, layer pullets, and meat turkey farms had older barns, in comparison to broiler or laying hen farms (Table 2). Structural damage, such as gaps or cracks in poultry barns, was reported by 44 % of participants (Table 3), and this was confirmed during farm visit observations. Impaired condition of barns was highest for broiler parent breeders and lowest for broiler farms, but the difference was not significant. All organic farms (*n* = 13) had poultry houses with a covered veranda between the barn and outdoor range. Due to an HPAI housing order, no birds had access to an outdoor range during the study period, but on three farms the birds had had access to a covered veranda, of which two later denied access when a HPAI restriction zone was established. Deterrents or other methods to reduce the number of wild birds on the farm were often applied (Table 3), and the most common method was hunting. At the visual inspection, additional variations in biosecurity beyond what was captured from the questionnaire and Biocheck were observed (Fig. 3).

**Table 3:** Selected biosecurity questions and results from 48 poultry farms in Sweden, based on questionnaire (Q) and Biocheck (B) or expanded from Biocheck (BE).

Biosecurity measure	n	%	Data-origin
Visitors and farmworkers			
Good anteroom layout^1^			
Yes	22	46	Q
No	26	54	
Number of hygiene barriers to be crossed between the outdoors and poultry areas			
None	1	2	Q
One	7	15	
Two	33	69	
Three	4	8	
Four	3	6	
Do visitors and farmworkers have to wear farm-specific shoes before they are allowed to enter poultry areas?			
Yes	48	100	BE
No	0	0	
Is a disinfection bath for boots used?			
Yes	1	2	BE
No	47	98	
Hand hygiene routines			
Washing with soap and water and disinfection	25	52	BE
Washing with soap and water	17	35	
Disinfection only	3	6	
No hand hygiene measure	3	6	
Locations where hand hygiene measures are carried out			
Both at farm and house hygiene lock	11	23	BE
At the farm hygiene lock	28	58	
At the house hygiene lock	6	13	
No hand hygiene measures	3	6	
Location for changing clothes			
Both at farm and house hygiene lock	7	15	BE
At the farm hygiene lock	29	60	
At the house hygiene lock	12	25	
Are there visible labels indicating different hygiene zones?			
Yes	3	6	Q
No	45	94	
Was showering required to enter the poultry house?			
Yes	11	23	Q
No	37	77	
Were dedicated footwear used in the transition zone between farm hygiene lock and house hygiene lock?			
Yes	35	73	Q
No	6	13	
Not applicable	7	15	
Depopulation			
What happens with the animals after their production cycle?			
Slaughtered in abattoir in Sweden	33	69	Q
Slaughtered in abattoir in another European country	6	13	
On-farm euthanasia	6	13	
Not applicable	3	6	
Do the driver and the catching team receive and wear farm specific or disposable clothes and footwear during the loading of poultry?			
Always	14	29	B
Never	28	58	
Sometimes	3	6	
Not applicable	3	6	
Feed water and bedding			
Was poultry given straw, hay, other roughage and/or supplements?			Q
Yes	22	46	
No	26	54	
Was straw, hay, other roughage and/or supplements stored in a clean space protected from rodents and wild birds?			
Yes	11	23	Q
No	11	23	
Not applicable	26	54	
Could straw, hay, other roughage and/or supplements be taken directly from the clean area to the poultry house without passing outdoors?			
Yes	3	6	Q
No	19	40	
Not applicable	26	54	
How were vehicles and/or equipment used to supply straw, hay or other roughage to poultry houses stored?			
Inside	9	19	Q
Outside	4	8	
Not applicable	35	73	
Manure and carcasses			
Is manure being stored on the farm?			
Yes	24	50	B
No	24	50	
Is the manure removed and disposed of appropriately through the dirty road?			
Yes	11	23	B
No	37	77	
What happens with the carcasses?			
The carcasses are burned	17	35	B
The carcasses are burned or collected by a rendering company	4	8	
The carcasses are stored and collected by a rendering company	27	56	
Can the carcasses be collected by the rendering company without entering the farm e. g. from the public road?			
Yes	19	40	B
No	12	25	
Not applicable	17	35	
Material supply			
Is there any material being shared with other farms that enters the poultry houses and or has contact with your poultry?			
Yes	6	13	B
No	42	88	
Are specific measures taken for the introduction of material (e.g. UV-disinfection unit, alcohol disinfection)?			
Yes	19	40	B
No	29	60	
Infrastructure and biological vectors			
Is the farm site divided into a clean and dirty area?			
Yes	6	13	B
No	42	88	
Does the poultry have access to the outside i.e. the open air?^2^			
Yes	12	25	B
No	36	75	
Is the farm fenced?			
It's only partially fenced	1	2	B
No	47	98	
Are vehicle disinfection baths or channels available at the entrance of the farm?			
Yes	0	0	B
No	48	100	
Is the outside of the farm around the walls paved and clean e.g. removal of weeds and waste?			
Yes, it's completely paved and clean	19	40	B
It's only partially paved and clean	29	60	
Are vermin, i.e. rats or mice considered to be a problem at the farm?			
Often	3	6	B
Sometimes	31	65	
Never	14	29	
Is a rodent control programme present on the farm?			
Yes	48	100	B
No	0	0	
What strategy was used for rodent control?			
Rodenticides are in permanent use	27	56	Q
Rodenticides are used if signs of rodents are noted	19	40	
NA	2	4	
Was there structural problems with the poultry building, such as damage, cracks and gaps?			
Yes	21	44	Q
No	27	56	
Were there measures to prevent wild birds on the farm?			
Yes, by making the farm unattractive to wild birds	3	6	Q
Yes, by using deterrents	34	71	
No	11	23	
Were insects present in poultry houses?			
Yes	22	46	Q
No	26	54	
Was the veranda bird and rodent-proof ?			
Yes	3	6	Q
No	10	21	
Not applicable	35	73	
Cleaning and disinfection			
Are the poultry houses cleaned after each production cycle?			
Yes	48	100	
No	0	0	
Is detergent added to water during cleaning?			
Always	23	48	B
Sometimes	6	13	
Never	19	40	
Egg management			
Are the eggs that are ready for transport stored in a specific storeroom i.e. in a room different from the egg room?			
Yes	23	48	B
Not applicable	25	52	
Does the driver have access to the egg facilities of the farm?			
No, the driver doesn't have access at all	1	2	B
Yes, but only to the specific storeroom	21	44	
Yes, the driver has access to both the egg room and specific storeroom	1	2	
Not applicable	25	52	
Are eggs being sold at the farm?			
Yes	10	21	B
No	13	27	
Not applicable	25	52	

The answers represent circumstances at the time of restrictions zones for HPAI in either 2020/2021 or 2021/2022 season.

1 Based on direction of movement through the changing room and shower, ease of access to and location of handwashing facilities in relation to separation of clean and dirty areas, space allowance for changing clothes and boots, design of the hygiene barrier and clarity as regards separation of clean and dirty areas.

2 The question was answered based on the farming system, not the situation during the HPAI restrictions and housing order.

**Table 4:** Examples of biosecurity measures with significant differences between poultry categories.

Biosecurity measure	Overall n (%)	Broiler parent breeder n (%)	Layer pullets n (%)	Laying hen n (%)	Broiler n (%)	Meat turkey n (%)	*p*-value^1^
Was a visitor’s log available at the time of the visit?							<0.001
Yes	22 (46)	9 (100)	2 (100)	7 (47)	2 (20)	2 (17)	
No	26 (54)	0 (0)	0 (0)	8 (53)	8 (80)	10 (83)	
Is there a strict separation between the clean and the dirty area of the house hygiene lock?							<0.05
Yes	37 (77)	3 (33)	2 (100)	11 (73)	10 (100)	11 (92)	
No	11 (23)	6 (67)	0 (0)	4 (27)	0 (0)	1 (8)	
Where does farmworkers change clothes?^2^							<0.001
Both at farm and house hygiene lock	7 (15)	0 (0)	0 (0)	5 (33)	2 (20)	0 (0)	
At farm hygiene lock	29 (60)	9 (100)	1 (50)	1 (7)	6 (60)	12 (100)	
At house hygiene lock	12 (25)	0 (0)	1 (50)	9 (60)	2 (20)	0 (0)	
Where does farmworkers wash hands?^2^							<0.05
Both at farm and house hygiene lock	11 (23)	1 (11)	0 (0)	4 (27)	6 (60)	0 (0)	
At farm hygiene lock	28 (58)	8 (89)	1 (50)	6 (40)	3 (30)	10 (83)	
At house hygiene lock	6 (13)	0 (0)	1 (50)	4 (27)	1 (10)	0 (0)	
No hand hygiene measure	3 (6)	0 (0)	0 (0)	1 (7)	0 (0)	2 (17)	
Do the driver and the catching team receive and wear farm specific or disposable clothes and footwear during the loading of poultry?							<0.05
Always	14 (29)	0 (0)	0 (0)	7 (47)	0 (0)	7 (58)	
Sometimes	3 (6)	0 (0)	0 (0)	2 (13)	1 (10)	0 (0)	
Never	28 (58)	8 (89)	0 (0)	6 (40)	9 (90)	5 (42)	
Not applicable	3 (6)	1 (11)	2 (100)	0 (0)	0 (0)	0 (0)	
Is manure being stored on the farm?							<0.001
Yes	24 (50)	0 (0)	2 (100)	14 (93)	2 (20)	6 (50)	
No	24 (50)	9 (100)	0 (0)	1 (7)	8 (80)	6 (50)	
Is there any material being shared with other farms that enters the poultry houses and or has contact with your poultry?							<0.001
Yes	6 (13)	0 (0)	0 (0)	0 (0)	6 (60)	0 (0)	
No	42 (88)	9 (100)	2 (100)	15 (100)	4 (40)	12 (100)	
Is detergent added to the water during cleaning?							<0.05
Always	23 (48)	7 (78)	1 (50)	4 (27)	7 (70)	4 (33)	
Sometimes	6 (13)	0 (0)	0 (0)	1 (7)	2 (20)	3 (25)	
Never	19 (40)	2 (22)	1 (50)	10 (67)	1 (10)	5 (42)	

1 Fisher’s exact test.

2 When only one hygiene lock was available it was categorized as farm hygiene lock.

### Farmers’ perceptions of viral introduction

The most likely introduction route(s) of HPAIV, as perceived by participants, were through ventilation openings (65 %), via farmworkers (29 %), via transports (13 %) and via rodents (8 %). Introduction through ventilation openings was mostly mentioned in the context of airborne spread from wild birds but also connected to the possibility of droppings from wild birds entering the barn, or airborne transmission from other poultry farms.

### Biosecurity improvements in response to HPAI epidemic

Most farms (79 %, 93 % of case farms and 73 % of non-case farms) reported having made improvements in biosecurity following the outbreaks. The measures included: enhanced adherence to hygiene routines at the hygiene locks, measures to avoid contamination of farmyards, improved hygiene for storage and delivery of bedding material, use of wild bird deterrents, more restrictive visitor access, improved visitor hygiene, establishment of color-coded hygiene zones, more frequent disinfection of floors in anteroom and egg storage, disinfection of materials brought into poultry barns, improved routines for handling and removal of dead birds, and regular meetings with farmworkers where biosecurity was discussed. Furthermore, 63 % of participants identified unaddressed biosecurity needs; many of these measures focused on preventing indirect virus exposure from wild birds.

### Biocheck scores

**Fig. 4:**
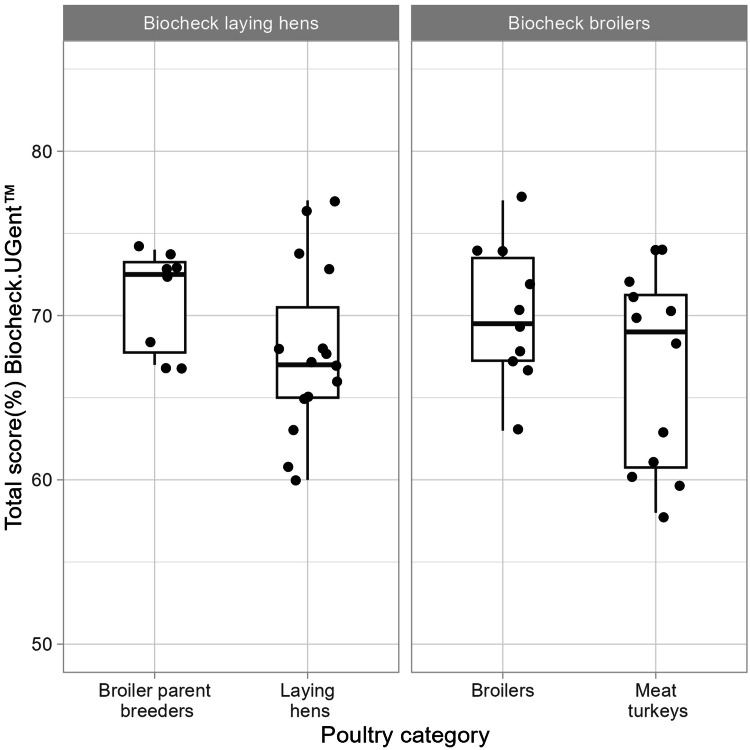
Total Biocheck score by poultry category in Sweden during 2020 and 2021 generated by the Biocheck scoring tool. The protocol for laying hens was used for broiler parent breeders and laying hens (left) and the protocol for broilers was used for broiler and meat turkey farms (right). The maximum Biocheck score is 100 %.

**Fig. 5:**
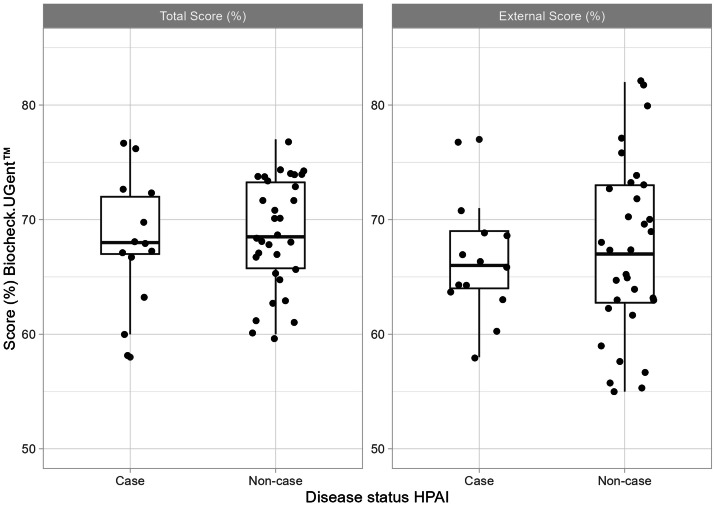
Total (left) and external (right) Biocheck score (%) by HPAI status in Sweden during 2020 and 2021 generated by the Biocheck scoring tool using the protocol for broiler farms (broiler and meat turkey farms) and laying hens (laying hen and broiler parent breeder farms). The maximum Biocheck score is 100 %.

The total score ranged from 58 % to 77 %, with a mean of 69 % (95 % CI: 67 % to 70 %; maximum 100 %). Breeder farms had higher total score than laying hen farms, and broiler farms had higher total score than turkey farms, although neither difference was significant (*p* = 0.08 and *p* = 0.15, respectively; Fig. 4). There was no significant difference in external or total score between case farms and non-case farms (Fig. 5).

There were no significant differences in total score between the three geographical regions, nor between the different farm size categories, or between organic and conventional farms. Farms with a higher number of birds had a higher total score, but this was not significant (*p* = 0.07). The total score was higher for farms with at least one woman among the interviewee(s) (*p* < 0.05). There was no significant difference in the total score before and after the HPAI outbreaks, the largest increase on an individual farm being 4 %.

**Fig. 6:**
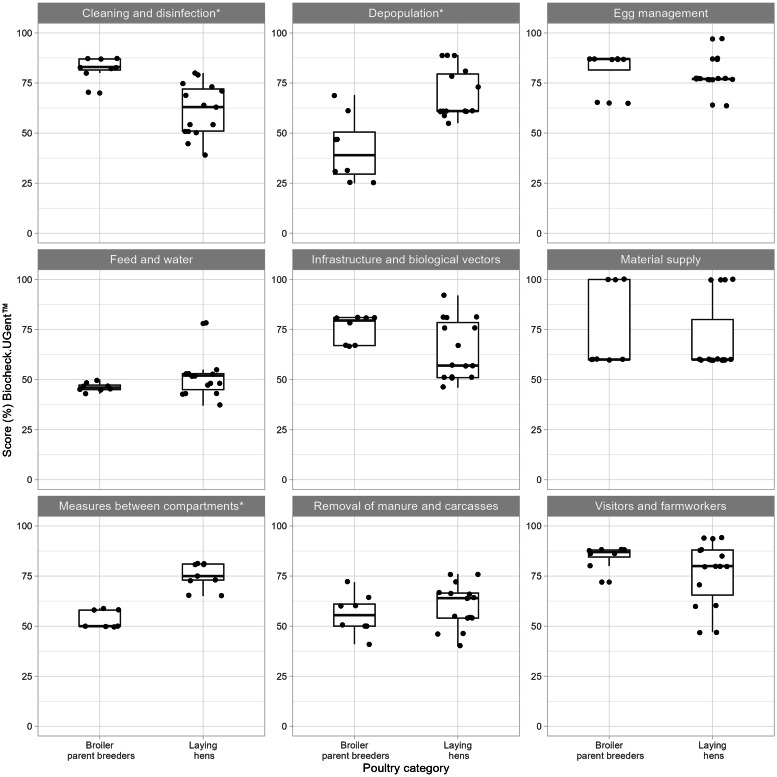
Scores from the Biocheck protocol for laying hens in the biosecurity subcategories: *cleaning and disinfection, depopulation, egg management, feed and water, infrastructure and biological vectors, material supply, materials and measures between compartments, removal of manure and carcasses* and *visitors and farmworkers,* for the poultry categories broiler parent breeders and laying hens. Asterisk (*) denotes a significant difference between groups (*p* < 0.05).

**Fig. 7:**
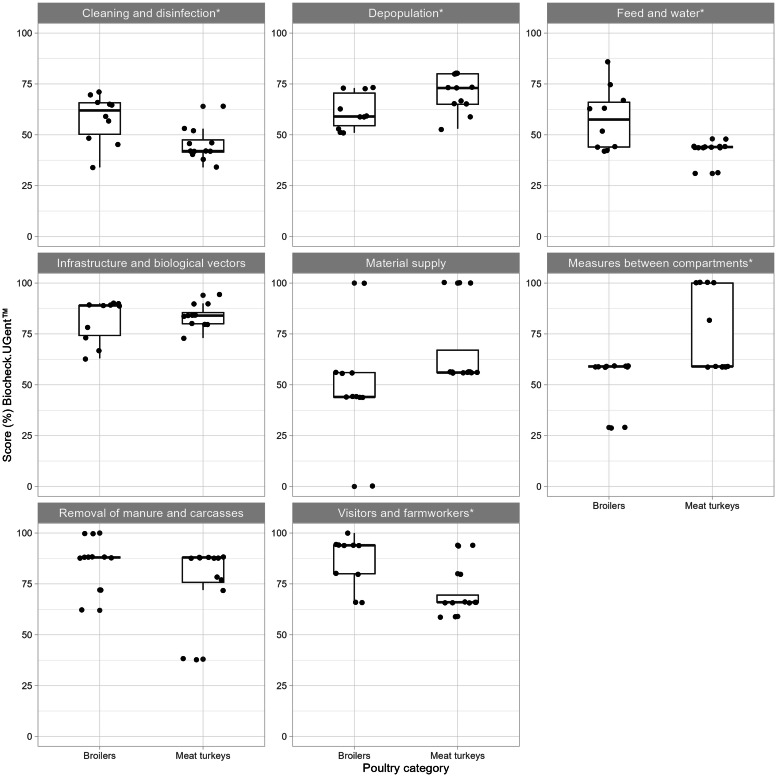
Scores from the Biocheck protocol for broilers in the biosecurity subcategories: *cleaning and disinfection, depopulation, feed and water, infrastructure and biological vectors, material supply, materials and measures between compartments, removal of manure and carcasses* and *visitors and farmworkers,* for the poultry categories broilers and meat turkeys. Asterisk (*) denotes a significant difference between groups (*p* < 0.05).

Scores for selected biosecurity subcategories are shown in Fig. 6 (laying hens and broiler parent breeders using the protocol for laying hens) and Fig. 7 (broilers and meat turkeys using the protocol for broilers).

### Qualitative analysis

The themes identified from approximately 300 unique free text comments and observations were knowledge, infrastructure, conflict of interest, non-compliance with routines, powerlessness, proportionality and risk-based approach, and heterogeneity.

The theme *knowledge* included both needs for specific knowledge on effective biosecurity measures against HPAI, and challenges on individual farms. While some interviewees appeared updated on biosecurity, others displayed knowledge gaps. Recruiting and keeping competent farmworkers was raised as a challenge by farmers. Furthermore, knowledge transfer within the farms was often stepwise, with only the farmer or a limited number of employees attending biosecurity courses, later sharing the knowledge. Translation was described as necessary with non-Swedish-speaking employees, and concern for misunderstanding was raised. Supportive material, i.e. fact sheets, available in different languages were requested.

The *farm infrastructure* was sometimes raised as a barrier to biosecurity implementation. In several cases, farmers were aware that the farm infrastructure was not ideal from a biosecurity perspective, but that the location, layout or condition of the farm buildings were factors they had to manage. Financial limitations or rental of the farm were raised as constraints hindering them from improving layout or condition of existing barns, or building new.

*Conflicts of interest* were present, specifically concerning the animal welfare implications of indoor poultry housing when the planned stocking density was based on access to a covered veranda. Other conflicts described included rules in organic production limiting the usage of effective disinfectants, and the requirement to provide roughage and substrate (i.e. sand) for dustbathing despite challenges in introducing this in a hygienic manner. Other difficulties mentioned were the strive to reduce the use of rodenticides, and provision of appropriate workwear for catching teams from occupational and biosecurity standpoints.

Several comments were expressed in relation to *non-compliance with routines*. These were often described as exceptions from normal routines and explanations were given such as the farm-specific clothes for visitors were being washed and were therefore not available, or a missing visitors' log was currently located elsewhere. Another example was comments from farmworkers indicating that rules were occasionally bent.

The theme *powerlessness* included factors described by the respondents to be beyond their control. Examples included the presence of rodents or wild birds, other professionals visiting the farm with limited respect for biosecurity routines, the location of other poultry farms nearby, and suboptimal farm infrastructure.

*Proportionality and risk-based approach* included several comments that described a risk-based approach with reinforced biosecurity during periods with increased risk of HPAI outbreaks and a more relaxed approach at other times. Another perspective of the interviewees’ risk awareness was reflected through comments related to proportionality, e.g. perceived low-risk introduction routes were pointless to address in view of the presence of higher risks that were not addressed. One example was questioning the benefits of washing hands before entering the barn, while roughage, unprotected from wild birds, was introduced several times per week.

*Heterogeneity* reflected comments related to which biosecurity measures were implemented, which were not, and perceptions on adequate levels of biosecurity. Notably, there was considerable variation in individual approaches to biosecurity practices, as well as different interpretations of some concepts, including the meaning of “clean”.

## Discussion

The recent global spread of HPAIV highlights the need for improved poultry farm biosecurity. In this study we investigated biosecurity measures applied on commercial farms with different poultry categories of chickens and turkeys during and after the worst avian influenza epidemic in Sweden in 2020–2021. Data were collected to assess biosecurity by both quantitative and qualitative methods.

Our study showed that several areas such as hand hygiene routines, management of roughage, infrastructure including anteroom layout, separation of clean and dirty outdoor areas, and training of farmworkers, could be improved. Infrastructure and barn maintenance are important to create barriers between poultry and wild birds (EFSA AHAW Panel (EFSA Panel on Animal Health and Welfare) More et al., 2017). Contrary to what was expected, broiler parent breeder farms had older barns (Table 2), sometimes in worse condition than barns used for other poultry categories. Broiler parent breeder farms also often had suboptimal biosecurity at the house hygiene lock. Recent studies (Laconi et al., 2023; Souillard et al., 2024) found higher biosecurity on breeder farms. Our study also indicated higher biosecurity in broiler parent breeders in some respects and a non-significant higher total score compared to laying hen farms. Broiler farms had newer barns in better condition and they also scored high on measures related to visitors and farmworkers. One explanation for this may be the longstanding efforts to minimize the prevalence of *Campylobacter* spp. (Lindqvist et al., 2022), with positive impact on the overall biosecurity. The condition of the barns is also relevant for rodent control, as rodents may introduce both HPAIV (Velkers et al., 2017) and other pathogens (Backhans and Fellström, 2012). Most participants reported that rodenticides are required permanently to control rodents, thereby creating a conflict of interest with the regulations inflicted to mitigate the risk of rodenticide resistance (European Parliament and Council of the European Union, 2012). Animal welfare was also a conflict of interest, as the efforts to prevent HPAI may lead to poultry being denied access not only to an outdoor range but also to a covered veranda that contributes positively to poultry welfare (EFSA AHAW Panel (EFSA Panel on Animal Health and Animal Welfare) et al., 2023b; EFSA AHAW Panel (EFSA Panel on Animal Health and Animal Welfare) et al., 2023c). Further, restricting access could impair animal welfare by increasing the stocking density.

Notably, a large variation in the level of biosecurity was observed between farms with category-specific strengths and challenges. The variation was unexpected as most farms in the study follow comprehensive industry biosecurity programs based on widely accepted principles. The qualitative analysis revealed several reasons for the implementation being suboptimal on some farms, which contributed to the observed heterogeneity. The use of qualitative methods has increased in veterinary medicine and proven informative to study complex multidisciplinary problems (Degeling and Rock, 2020) to approach the “why”. The findings in the qualitative analysis fit well with a recent study from the UK (Hosseini et al., 2025), thus indicating that challenges to implement biosecurity may be similar also in other countries. The qualitative analysis also found a need for knowledge support, consistent with a study by Gröndal et al. (2023) in which farmers expressed that measures should have proven efficacy to motivate costs. Previously, the effect of measures such as best practices for hand hygiene (Racicot et al., 2013), and limitation of footbaths as a stand-alone measure (Hauck et al., 2017), have been highlighted, and similar approaches to a wider range of preventive measures, including HPAIV-specific prevention, were called for by the participants in our study. Knowledge gaps do exist, but it can be assumed that no single measure is 100 % effective. As mechanical transmission of pathogens may occur through a sequence of events (Dee et al., 2002), a series of preventive measures are therefore warranted. This practice did not seem to be established among participants in our study however. For example, many farms did not fully utilize all existing hygiene locks. Additionally, many farms did not separate clean and dirty areas outdoors.

We were not able to demonstrate a higher biosecurity level in non-case farms compared to case farms. This may suggest that outbreaks are more associated with environmental contamination from wild birds than to breaches in biosecurity. However, the study did show that breaches occur, and with higher biosecurity across the industry, some outbreaks might have been prevented. Another factor was the differing response rates, as case farms were more inclined to participate than non-case farms, which may have biased comparisons between the two groups.

Moreover, capturing biosecurity practices is often challenging and we chose farm visits for data collection, as telephone interviews previously have provided limited information (Eriksson et al., 2019). The visits were valuable in providing an understanding of the range of practices implemented and different levels of biosecurity not captured by a “yes” or “no” answer. They also enabled a validation of the interview responses, as demonstrated by Nespeca et al. (1997). All visits were conducted by the same person, which minimized the risk of interviewer bias, but interviewer effects may still have influenced the participants' responses. A known limitation with face-to-face interviews is the risk of social desirability bias where good practices are overreported compared to bad practices (de Leeuw, 2018). To fully capture what the farmworkers do, other methods such as video recording may be required. Based on such work done by Racicot et al. (2011) and recently (Elbers and Gonzales, 2025), we can assume that people do not always comply with proclaimed biosecurity routines.

The farms were selected for dual purposes: 1) to assess biosecurity practices (present study) and 2) to investigate risk factors for HPAI outbreaks (Grant et al. in preparation). As a result, there is a risk of selection bias as the farms were not randomly sampled and may therefore not be a true representation of the target population. While there could potentially be higher biosecurity in areas without reported HPAI outbreaks, available data show that regions with HPAI outbreaks in poultry overlapped with those where HPAI cases in wild birds were reported (EFSA European Food Safety Authority, ECDC European Centre for Disease Prevention and Control, EURL European Reference Laboratory for Avian Influenza et al., 2021; Grant et al., 2022; Stiles et al., 2024). Earlier risk factor studies have also suggested that HPAI outbreaks were associated with presence of environmental contamination from waterfowl, rather than local or regional differences in biosecurity among poultry farms (Green et al., 2023; Patyk et al., 2023; Jensen et al., 2025).

Farms were represented by farmers or farmworkers. Listening to the perspectives from these key stakeholders gave a broader understanding of the biosecurity challenges. Another study proposed that research and interventions should focus more on farmworkers (Moya et al., 2025), and our study supports this. Not least because biosecurity training of farmworkers was identified as a key area for improvement. On the seven farms where all farmworkers had undertaken training only Swedish was spoken, which suggests that linguistic limitations may be one reason for the lack of training. The finding suggests that there is a need to offer different ways to train farmworkers, accounting for language barriers.

Standardized questionnaires, checklists, or scoring tools are commonly used to assess biosecurity in livestock production (Gelaude et al., 2014; Tilli et al., 2022). Biocheck offered a well-established methodology to assess on-farm biosecurity based on prioritization and weighing of measures according to their sector-specific relative importance for disease transmission (Gelaude et al., 2014). The participants also welcomed the opportunity to obtain a biosecurity assessment and as a result, some farms initiated biosecurity improvements. Our study aimed to compare biosecurity between poultry categories, however, Biocheck scores are not intended for this purpose and may potentially be misleading (Prof. J. Dewulf, Chair of Biocheck.Gent BV, Ghent, Belgium, personal communication). Instead, the comparisons between poultry categories were primarily focused on individual biosecurity measures, and scores were only compared when using the same protocol and was interpreted with caution (Fig. 6, Fig. 7). Using mismatched protocols, without the species-specific risk weighting, reduces the validity for comparing scores but was beneficial when comparing individual variables. Despite multiple improvements in biosecurity following the outbreaks, no significant increase in total scores was found, similar to a study by Tilli et al. (2024) using virtual farm tours and coaching group-discussions. In contrast, Caekebeke et al. (2021) demonstrated an increase in both external and internal scores after coaching broiler farmers. In the present study, it cannot be excluded that there wasn’t enough power to detect such differences. It’s also possible that the improvements made were not captured and rewarded by the risk-based scoring system used by Biocheck. The scores across the farms were also consistently well below 100 %, partially explained by that vehicle or boot disinfection baths were very uncommon. Disinfection baths are not part of the poultry industry biosecurity programs applied in Sweden, as disinfection baths require prior cleaning to remove organic matter, set contact time, and also frequent replacement of the disinfectant solution to remain effective (Stringfellow et al., 2009). In summary, while the use of Biocheck in our study design involved several tool limitations, it also offered important advantages.

Viral introduction through ventilation openings was considered as the most likely route of entry by our interviewees, which is similar to the study by Hosseini et al. (2025). This was not supported in a risk assessment from the Netherlands in which airborne HPAIV transmission from wild birds was deemed unlikely (de Vos and Elbers, 2024). Other studies have shown that DNA from waterbirds and particle matter can enter poultry barns through ventilation inlets (Elbers et al., 2022; Bossers et al., 2024). More research is clearly needed into the relevance of airborne transmission from wild birds to poultry in HPAI epidemiology. A strong belief in airborne transmission among farmers and farmworkers may give a feeling that preventing HPAI is beyond their control. This links to the theme powerlessness that was found in the qualitative analysis, in agreement with a study on German pig-farmers' decision-making to control African swine fever (Klein et al., 2023) and also in the context of viral diarrhea in cattle (Nöremark et al., 2016). When advising farmers, it is important to stress that a lot can be done to reduce the probability of viral introduction. Although the risk cannot be reduced to zero, implementing multiple risk-reducing measures, addressing the range of potential introduction routes, can significantly reduce the overall risk.

## Conclusion

The results of our study show a variation in the implementation of biosecurity on Swedish poultry farms affected by multiple factors, which highlights the need for tailored farm-specific biosecurity measures. There are multiple barriers to biosecurity implementation, and different interests need to be balanced. More research should be focused on what is effective and what is less effective and consider the feasibility, sustainability and costs of the respective biosecurity measures.

## Declaration of AI and AI-assisted technologies in the writing process

During the preparation of this work the authors used ChatGPT (OpenAI, 2025) in order to improve the draft readability and language. After using this tool/service, the authors reviewed and edited the text as needed and take full responsibility for the content of the publication.

## CRediT authorship contribution statement

**Malin Grant:** Conceptualization, Data curation, Formal analysis, Methodology, Visualization, Writing – original draft, Writing – review & editing. **Désirée S. Jansson:** Conceptualization, Methodology, Writing – original draft, Writing – review & editing. **Arianna Comin:** Conceptualization, Formal analysis, Methodology, Visualization, Writing – review & editing. **Magdalena Jacobson:** Conceptualization, Methodology, Writing – review & editing. **Maria Nöremark:** Conceptualization, Funding acquisition, Methodology, Project administration, Writing – original draft, Writing – review & editing.

## Disclosures

The authors declare that they have no known competing financial interests or personal relationships that could have appeared to influence the work reported in this paper.

## Appendix Supplementary materials

Supplementary material 1: Questionnaire HPAI biosecurity and risk factors Image, application 1

Supplementary material 2: Biocheck Broilers_EN Image, application 2

Supplementary material 3: Biocheck Laying hens_EN Image, application 3
