# Supplementary material for: Strengths, challenges, and variations - insights into biosecurity practices in Swedish poultry production following HPAI outbreaks
Source: Poult Sci. 2025 Sep 19;104(12):105871. doi: 10.1016/j.psj.2025.105871 (PMC12529493; doi:10.1016/j.psj.2025.105871)
Supplement: Supplementary file 1 — Supplementary material 1: Questionnaire HPAI biosecurity and risk factors [file mmc1.docx]

Questionnaire for farm visits

This is a translation of the original Swedish version used for interviews and on-farm observations.

The questions relate to conditions that prevailed during the peak season for avian influenza in 2020/2021 or 2021/2022 [referred to a “relevant period” below, and changes made after this period as specified in the questions]

Definitions:

Flock= Corresponds to an epidemiological unit, i.e. group of poultry with a separate compartment and hygiene lock.

IP = infected premise

Introduction to interviewees: The questions consist of two parts. A questionnaire with focus on avian influenza risk and Biocheck which focuses on biosecurity in general.

Observation points: farm yard, anteroom including hygiene locks, egg packing room, poultry barn from the outside including air vents, feed storage/silos, roughage storage, veranda and pasture, manure storage, carcass storage.

Basic information to be filled in in advance

| IP number and season. For non-case farms, the IP that caused the restriction zone in which the farm was included: |  |
| --- | --- |
| Animal owner and company name: |  |
| Name and role of the person being interviewed: |  |
| Sex of the person being interviewed: |  |
| Holding number [unique identifier of farm]: |  |
| E-mail address: |  |
| Telephone number: |  |
| Date of visit: |  |
| Date of outbreak or declaration of restriction zone: |  |

|  | Case farm: How do you think the avian influenza got into your poultry? What is the most likely route of introduction in your view?  Non-case farm: What was the risk of infection that worried you the most during the ongoing avian influenza outbreak in the area? If you would have had an outbreak, how do you think the virus would have gotten into your poultry? What would have been the most likely route of introduction? |
| --- | --- |
|  | Have there been any changes in biosecurity during or after the outbreak/restriction zones for avian influenza? If so, what changes have been made? |
|  | Who gives you advice or who do you ask for advice on biosecurity on your farm? (The question concerns farm-specific advice in addition to general recommendations) |
|  | Have you, or the person who gives you biosecurity advice, identified any specific measures that you should implement to further strengthen biosecurity? If so, which one(s)? Why haven't the measures been put in place yet? |

General "farm characteristics"

|  | How many flocks (epidemiological units) were there on the farm during the relevant period? |  |
| --- | --- | --- |
|  | Which species of poultry were kept? Also indicate the number of flocks for each species and indicate which species was first affected by HPAI (if applicable). | - Chickens - Turkeys - Miscellaneous, what: |
|  | Type of production during the relevant period?  Also, state the number of flocks for each type of production. | - Egg production - Meat production - Breeding (P) laying hens - Breeding (P) meat production - Layer pullets |
|  | Sex of turkeys (applies to turkeys only)  Also, enter the number of flocks for each sex. | - Hens - Cocks - Both |
|  | What was the age of the turkeys at the time of the outbreak/declaration of the restriction zone? (Turkeys only) |  |
|  | Number of poultry during the relevant period.  If there are multiple flocks, enter the number of birds per flock. |  |
|  | Enrolled in biosecurity programs? | - Yes, with *Swedish Egg Association* - Yes, with *Swedish Poultry Meat association* - Yes, other than the above - No |
|  | Organic production | - Yes - No - Partly |
|  | Member of *Swedish Egg Association* | - Yes - No |
|  | Member of *Swedish Poultry Meat Association* | - Yes - No |
|  | Does the farm have a written farm-specific biosecurity plan? | - Yes - No - Don't know |

Farmworker and visitors

|  | Have the farmer and/or farmworkers received training in biosecurity in the last five years before the relevant period? (Question relates to all employees and the farmer). | - Yes - No - Some have been trained - Don't know |
| --- | --- | --- |
|  | Were instructions on biosecurity routines posted in written or visual form at the facility during the relevant period?  Describe what instructions were available, in what format and in which language.  Also to be observed on site. | - Yes - No |
|  | Did you experience any challenges or difficulties in communication in your daily work due to language barriers during the relevant period? | - Yes, often - Sometimes - Not at all - Don't know |
|  | How many different languages were spoken in the daily work on the farm during the relevant period? | - 1 - 2 - 3 - 4 or more |
|  | Did all farmworkers have access to instructions (oral or written) in their mother tongue or another language that they master well? | - Yes - No - Don't know |

Slaughter

|  | What happens with the animals after their production cycle? | - Slaughtered in abattoir in Sweden - Slaughtered in abattoir in another European country - On-farm euthanasia - Not applicable |
| --- | --- | --- |

Egg handling

|  | Was there a registered egg packing centre on the farm during the relevant period? | - Yes - No |
| --- | --- | --- |
|  | If there was no registered egg packing centre, which packing centre handled the eggs during the relevant period? |  |

Infrastructure and biological vectors

|  | Have you checked and/or observed faults/damage to the integrity of the buildings (refers to poultry barn) such as damage, cracks, gaps in ceilings, walls, windows or doors during the relevant period?  Also to be observed on site. | - Yes, there are damage - No, no damage noted - Don't know if there is damage |
| --- | --- | --- |
|  | Was there moss, leaves or other organic material on the roof of any poultry barns during the relevant period?  Also to be observed on site. | - Yes - No - Don't know |
|  | Were poultry barns heated during the relevant period? | - Yes - No - Don't know |
|  | What was the lowest temperature in the poultry areas during the relevant period? |  |
|  | Type of ventilation?  Also specify the number of flocks for each option. Also indicate which option represents the flock first infected with HPAI (if applicable). | - Negative pressure ventilation - Balanced ventilation - Natural ventilation - Other, what? ___________________ |
|  | Placement of air inlets?  If more than one option, specify the number of flocks for each placement category. Also indicate which option represents the flock first infected with HPAI (if applicable).  Also to be observed on site. | - On the roof - In the walls - Via the loft - Supply air ceiling - Other, what?   ___________________ |
|  | Placement of air outlets?  If more than one option, specify the number of flocks for each placement category. Also indicate which option represents the flock first infected with HPAI (if applicable).  Also to be observed on site. | - On the roof - In the walls - Other, what?   ____________________ |
|  | Protection of air inlets?  If more than one option, specify the number of flocks for each option. Also indicate which option represents the flock first infected with HPAI (if applicable).  If there is variation within a flock, the question should be answered based on the "lowest level".  Also to be observed on site. | - Nets - Hoods/covers - Drip trays - Anti-bird spikes - Other, what?   ___________________ |
|  | Protection of air outlets?  If more than one option, specify the number of flocks for each option. Also indicate which option represents the flock that was first infected with HPAI (if applicable).  If there is variation within a flock, the question should be answered based on the "lowest level".  Also to be observed on site. | - Nets - Hoods/covers - Drip trays - Bird spikes - Other, what?   ___________________ |

Feed, water and bedding:

|  | Was non heat-treated grain used during the relevant period?  *If no/don't know, go to question 34.* | - Yes - No - Don't know |
| --- | --- | --- |
|  | If yes, do you know how the grain has been handled? | - Yes, in closed systems - Yes, open handling may have occurred - No, I don't know |
|  | Did you use straw, hay or other roughage or supplements?  How often was straw, hay, other roughage and/or supplements introduced during the relevant period?  *If not used, go to question 38.* | - Not used - Daily - Once a week or more often - Regularly during the current production cycle, but less often than once a week - Only if necessary, on occasional times - Only before each new production cycle - Don't know |
|  | Was straw, hay, other roughage and/or supplements stored in a clean space protected from rodents and wild birds during the relevant period?  Also to be observed on site. | - Yes, protected by building - Yes, wrapped bales - No - Don't know |
|  | Could straw, hay, other roughage and/or supplements be taken directly from the storage area to the poultry area without passing outdoors during the relevant period?  Also to be observed on site. | - Yes - No - Don't know |
|  | How were vehicles and/or equipment (e.g. bale grapples/bale spikes) stored and handled during the relevant period? This question refers to equipment used to introduce material to poultry barns.  Also to be observed on site. | - They were kept indoors in a space protected from wild birds - They were kept under a roof or indoors, but wild birds could fly in. - They were stored outdoors - Don't know |
|  | How often was new litter, other than straw, added to the poultry area during the relevant period? | - Not used - Daily - 1-6 times per week - Regularly, but less often than once a week - Only before each new production cycle - Don't know |

|  | How were the poultry housed during the relevant period?  If it differed between flocks, specify the number of flocks for each type. Also indicate which option represents the flock first infected with HPAI (if applicable). | - Indoor only - Indoor + veranda - Indoor + veranda + outdoor pasture - Indoor + outdoor pasture |
| --- | --- | --- |
|  | If there was a veranda, was it bird and rodent-proof during the relevant period? If different, specify the number of flocks for each option.  Also indicate which option represents the situation for the flock first infected with HPAI (if applicable).  Also to be observed on site. | - Yes - No - Don't know |

Location of the farm

|  | The farm is located in:  To be observed on site. | - Plains/arable land - Mixed countryside - Forested area - Don't know |
| --- | --- | --- |

|  | To what extent and in what way were waterfowl (geese, ducks or swans) present in the vicinity of the farm (within 500 m) during the relevant period? | - No waterfowl observed - Seen in tens - Seen in hundreds or more - Seen in thousands of birds   --------------------------------   - Were seen sporadically (a few days) - Were seen frequently or for extended periods (weeks or more)   ---------------------------------   - Flew over the farm - Grazed/rested in the fields |
| --- | --- | --- |
|  | To what extent were bird species other than waterfowl seen on the farm during the relevant period? | - None observed - Single birds - Seen in tens - Seen in hundreds or more   ---------------------------------   - Seen sporadically (a few days) - Were seen frequently or for extended periods (weeks or more) |
|  | Where on the farm were the birds observed? | |
|  | Of the birds noted on the farm itself, did you recognize any of the species and can you name any? | |
|  | Did wild birds enter the poultry barns during the relevant period? | - Yes - No - Don't know |
|  | Was any kind of measure used to keep wild birds away from the farm during the relevant period?  Also to be observed on site. | - Yes, a deterrent method - Yes, anti-bird spikes - Yes, changes to make the environment unattractive to wild birds - No - Don't know |
|  | If yes, what measures were applied? | |
|  | Describe your perceived impact of the different measures to keep the wild birds away from the farm: | |
|  | Were there wild boars on and/or around the farm during the relevant period (within 1 kilometre)? | - Yes - No - Don't know |
|  | Indicate which insects/parasites were present in the poultry area during the relevant period. | - Manure beetles or other beetles - Fruit flies/ hoverflies - Poultry red mite - Other, species? _____________ - No insects/parasites were noted |

|  | Were crops grown or was there a fruit orchard on the land closest to the farm during the relevant period?  Also to be observed on site. | - Yes - No - Don't know - If so, what was grown on the land? |
| --- | --- | --- |
|  | Were there any activities using machines, vehicles or people on the land closest to the farm during the relevant period? If yes, please describe the activity and the approximate time when it usually took place?  Examples: Harrowing, ploughing, harvesting, manure spreading, hunting, release of farmed wild birds  *If no/don't know, go to question 55*. | - Yes - No - Don't know   What activity?  Approximate time point? |
|  | If activities occurred on nearby land, could the same machines, vehicles or people drive/walk on surfaces used for poultry production? | - Yes, on surfaces on the farm - Yes, on the farm's access road - No - Don't know |
|  | Type of access road to the farm?  Also to be observed on site. | - Asphalt road - Gravel road |
|  | Did anyone other than you or transports connected to your production use the access road? | - Yes - No |

Finally

|  | Is there anything else you would like to add to the questions or anything else that was not covered in the questionnaire? |
| --- | --- |

Additional questions/observation points to complement Biocheck

|  | Good layout in the anteroom?  Observations on farm: Direction of movement through the changing room and shower, ease of access to and location of handwashing facilities in relation to separation of clean and dirty areas, space allowance for changing clothes and boots, design of the hygiene barrier and clarity as regards separation of clean and dirty areas. |
| --- | --- |
|  | Were different hygiene zones in the facility clearly marked by labels/signs? |
|  | Number of hygiene barriers to be crossed between outdoors and poultry areas? |
|  | Were farmers/farmworkers/visitors required to shower before entering poultry areas? |
|  | Were dedicated footwear used in the buffer zone between outer (farm) and inner (house) hygiene locks? |
|  | What strategy was used for rodent control? |
