# Supplementary material for: Strengths, challenges, and variations - insights into biosecurity practices in Swedish poultry production following HPAI outbreaks
Source: Poult Sci. 2025 Sep 19;104(12):105871. doi: 10.1016/j.psj.2025.105871 (PMC12529493; doi:10.1016/j.psj.2025.105871)
Supplement: Supplementary file 2 — Supplementary material 2: Biocheck Broilers_EN [file mmc2.pdf]

FACULTY OF VETERINARY MEDICINE
accredited by EAEVE

biocheck.ugent

Version of Biocheck accessed in December 2021 and used for study: Grant et al., 2025, Strengths, challenges and variations - insights into biosecurity practices in Swedish poultry production following HPAI outbreaks

BIOCHECK POULTRY

Broilers

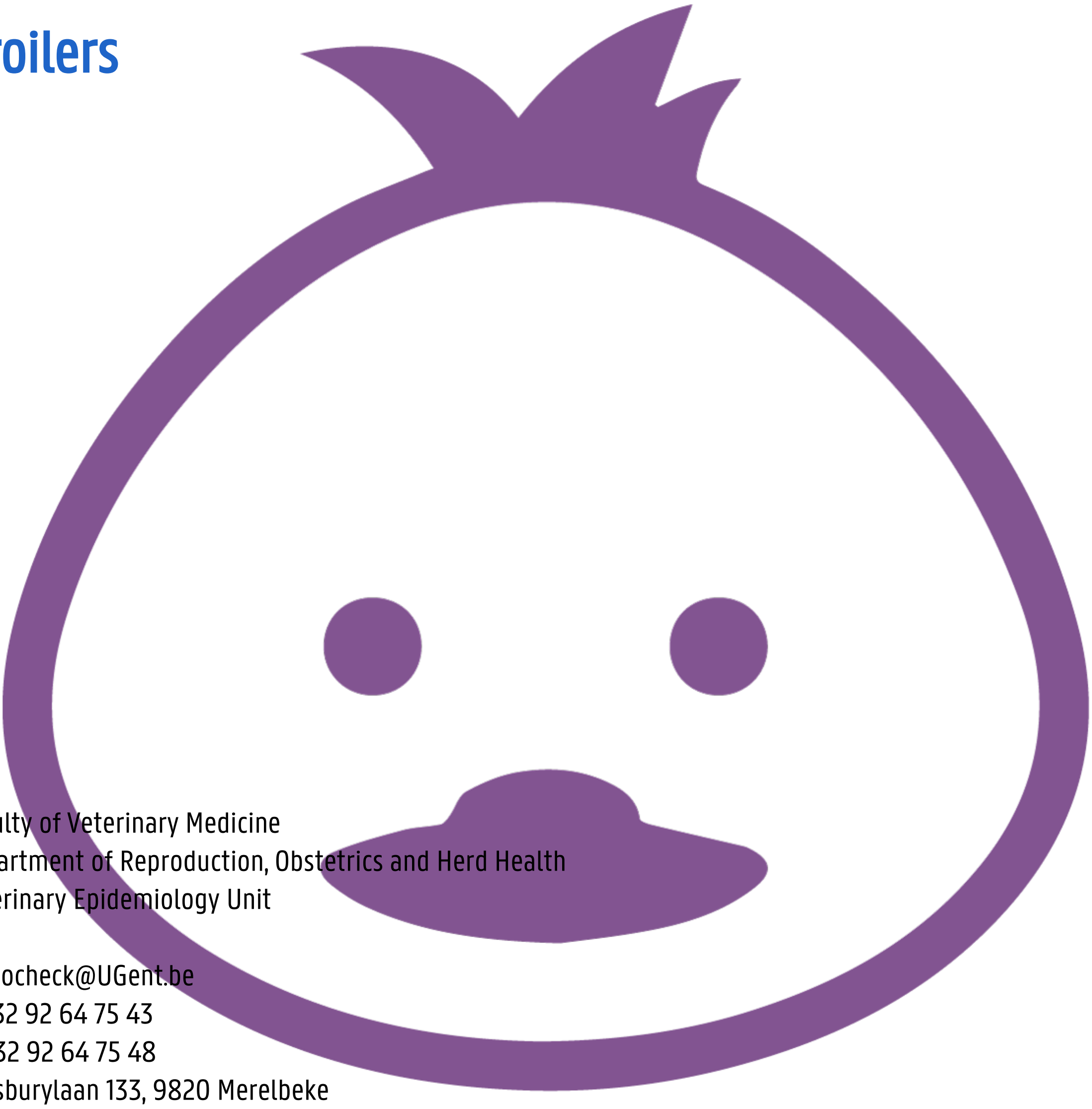

Faculty of Veterinary Medicine
Department of Reproduction, Obstetrics and Herd Health
Veterinary Epidemiology Unit

E: Biocheck@UGent.be
T: +32 92 64 75 43
+32 92 64 75 48
Salisburylaan 133, 9820 Merelbeke
Belgium

www.biocheck.ugent.be

GHENT UNIVERSITY

˜. Farm characteristics

I. How many years of experience in keeping poultry does the person in charge have?

.... years

II. How many people are working on the farm?

.... persons

III. How old (in years) is the oldest building in which poultry is being kept?

.... years

IV. How old (in years) is the newest building in which poultry is being kept?

.... years

A. Purchase of one-day-old chicks

1. Are your one-day-old chicks (during the last 2 years) always bought from the same original source? *(required)*

Select one option.
○ Always the same supplier
○ Sometimes a different supplier

2. Are the bought one-day-old chicks first delivered at your farm, i.e. before other farms are supplied by the same transport vehicle? *(required)*

Select one option.
○ Always
○ Sometimes
○ Never

3. Are the transport vehicles (including the transport crates and containers) cleaned and disinfected before the one-day-old chicks are loaded? *(required)*

Select one option.
○ Yes
○ No

4. How often a year are one-day-old chicks delivered to your farm? *(required)*

Select one option.
○ Less than 3 times a year
○ Between 3 and 6 times a year
○ More than 6 times a year

B. Depopulation of broilers (slaughterhouses, traders, individuals)

5. Is the transport vehicle (including the transport crates and containers) for poultry empty on arrival at the farm? *(required)*

Select one option.
○ Always
○ Sometimes
○ Never

6. Is the transport vehicle (including the transport crates and containers) for poultry always cleaned and disinfected on arrival at the farm? *(required)*

Select one option.
○ Yes
○ No

7. Do the driver and the catching team receive and wear farm-specific or disposable clothes/footwear during the loading of poultry? *(required)*

Select one option.
○ Always
○ Sometimes
○ Never

8. Are individuals and traders allowed to enter the poultry houses where direct contact with the poultry is possible? *(required)*

Select one option.
○ Always
○ Sometimes
○ Never

9. In how many steps does the depopulation of a poultry house take place? *(required)*

Select one option.
○ In one step
○ In two steps
○ In more than two steps

10. How often a year are broilers moved from the farm? *(required)*

Select one option.
○ Less than 6 times a year
○ Between 6 and 12 times a year
○ More than 12 times a year

C. Feed and water

11. Is the farm site divided into a clean and dirty area? *(required)*

The clean road/area is the area of the production site with restricted access, i.e. this is the area where only animals from the farm, persons after they have applied the hygienic measures in the hygiene lock, and farm-specific materials and vehicles are allowed. The dirty area comprises all other parts of the farm where visitors, external vehicles, ... have access to. The dirty area also includes the carcass storage facility.

Select one option.

○ Yes
○ No (Go to question 14)
○ I don't know (Go to question 14)

12. Is there a clear separation between the clean and the dirty area of the farm premises? *(required)*

Select one option.

○ Yes
○ No

13. Can the feeding company fill up the silos/deliver feed without entering the clean area? *(required)*

Select one option.

○ Yes
○ Only some of them
○ None

14. Does the feed supplier have access to the houses where direct contact with the poultry is possible? *(required)*

Select one option.

○ Always
○ Sometimes
○ Never

15. Are the feed silos or the feed storage rooms (storage of complete feed or concentrate) completely sealed against water, birds and vermin? *(required)*

Select one option.

○ Yes
○ No

16. How often a year does the feeding company fill up the silos or deliver feed? *(required)*

Select one option.

○ Less than 20 times a year
○ Between 20 and 35 times a year
○ More than 35 times a year

17. How often are bacteriological analyses of the drinking water performed? *(required)*

Select one option.

○ At least once a year
○ Every two years
○ Less frequent than every two years
○ Never (Go to question 19)

18. Where are the water samples for the bacteriological analyses taken? *(required)*

Select one option.

○ At the source
○ At the last drinker
○ At both locations, i.e. at the source and the last drinker

D. Removal of manure and carcasses

19. Is the manure removed and disposed of appropriately through the dirty road? *(required)*

The clean road/area is the area of the production site with restricted access, i.e. this is the area where only animals from the farm, persons after they have applied the hygienic measures in the hygiene lock, and farm-specific materials and vehicles are allowed. The dirty area comprises all other parts of the farm where visitors, external vehicles, ... have access to. The dirty area also includes the carcass storage facility.

Select one option.
○ Yes
○ No

20. Is there a separate carcass storage? *(required)*

Select one option.
○ Yes
○ No (Go to question 25)

21. Can the carcasses be collected by the rendering company without entering the farm premises (e.g. from the public road)? *(required)*

Select one option.
○ Yes
○ No

22. Is the carcass storage space protected from vermin, cats and/or dogs? *(required)*

Select one option.
○ Yes, it's completely protected
○ It's only partially protected
○ No

23. Is this carcass storage space cleaned and disinfected after each use? *(required)*

Select one option.
○ Always
○ Sometimes
○ Never

24. Is the carcass storage cooled? *(required)*

Select one option.
○ Yes
○ No

25. Are carcasses manipulated with gloves, or are hands cleaned and disinfected after manipulation of carcasses? *(required)*

Select one option.

○ Always
○ Sometimes
○ Never

E. Visitors and farmworkers

26. Are visitors obliged to notify you of their presence before entering the poultry houses (e.g. visitor's register)? *(required)*

Select one option.
○ Yes
○ No

27. Do all farmworkers (including the farm owner) abide by the access rules? *(required)*

Select one option.
○ Always
○ Sometimes
○ Never

28. Is a poultry-free period (longer than 12 hours) expected of all visitors before they are allowed to enter the poultry houses? *(required)*

Select one option.
○ Yes
○ No

29. Do visitors and farmworkers have to wear farm-specific clothing before they are allowed to enter the poultry houses? *(required)*

Select one option.
○ Yes
○ No

30. Do visitors and farmworkers have to wear farm-specific shoes/overshoes before they are allowed to enter the poultry houses? *(required)*

Select one option.
○ Yes
○ No

31. Do visitors and farmworkers have to wash and disinfect their hands before they are allowed to enter the poultry houses? *(required)*

Select one option.
○ Yes
○ No

32. How many times per year is access to the poultry houses granted to visitors? *(required)*

Select one option.

○ Access is never granted
○ Access is granted, but less than 12 times a year
○ Access is granted more than 12 times a year

33. Are there any farmworkers who also keep poultry or any other type of bird at home? *(required)*

Select one option.

○ Yes
○ No

34. Are there any farmworkers who also work on other poultry farms? *(required)*

Select one option.

○ Yes
○ No

F. Material supply

35. Is there any material being shared with other farms that enters the poultry houses and/or has contact with your poultry? *(required)*

Select one option.

○ Yes
○ No

36. Are specific measures taken for the introduction of material (e.g. UV-disinfection unit, alcohol disinfection)? *(required)*

Select one option.

○ Yes
○ No

G. Infrastructure and biological vectors

37. Does the poultry have access to the outside, i.e. the open air? *(required)*

Select one option.
○ Yes
○ No

38. Is manure being stored on the farm? *(required)*

Select one option.
○ Yes
○ No

39. Can wild birds enter the poultry houses? *(required)*

Select one option.
○ Yes
○ No

40. Are bird- and vermin-proof grids placed on the air inlets? *(required)*

Select one option.
○ Yes
○ No

41. Is the farm fenced? *(required)*

Select one option.
○ Yes, it's completely fenced
○ It's only partially fenced
○ No

42. Is the outside of the farm (around the walls) paved and clean (e.g. removal of weeds, waste, ...)? *(required)*

Select one option.
○ Yes, it's completely paved and clean
○ It's only partially paved and clean
○ No

43. Are vermin (i.e. rats, mice, etc.) considered to be a problem on the farm? *(required)*

Select one option.
○ Often
○ Sometimes
○ Never

44. Is a rodent control programme present on the farm (other than cats)? *(required)*

Select one option.
○ Yes
○ No

45. Do pets have access to the poultry houses (including the hygiene lock)? *(required)*

Select one option.
○ Yes
○ No

46. Is “backyard”-poultry or any other type of bird being kept on the farm premises? *(required)*

Select one option.
○ Yes
○ No

47. Are any other farm animals being kept on the same farm site? *(required)*

Select one option.
○ Yes
○ No

H. Location of the farm

48. Is there stagnant or running water within a 1-kilometre radius (0.6 miles) of the farm? *(required)*

Select one option.
○ Yes
○ No

49. At what distance (straight-line) is the nearest neighbouring poultry farm located? *(required)*

Select one option.
○ Less than 500 metres (Less than 0.3 miles)
○ Between 500 metres and 1 kilometre (between 0.3 and 0.6 miles)
○ More than 1 kilometre (more than 0.6 miles)

50. Is manure from other poultry farms spread on the neighbouring farmlands (within a 500-metre (0.3 miles) radius)? *(required)*

Select one option.
○ Often
○ Sometimes
○ Never

51. Does animal transport frequently occur (i.e. minimum once a day) via the public road (road less than 100 metres (328 feet) from your farm) where your farm is located at (e.g. due to the location of a slaughterhouse in the neighbourhood...)? *(required)*

Select one option.
○ Yes
○ No

I. Disease management

52. Is there a protocol for vaccinations? If so, do you always abide by it? *(required)*

Select one option.
○ Yes
○ No

53. Is there a regular (i.e. at least once a year) evaluation made of the disease status of the farm (e.g. serology, trends in slaughterhouse findings, etc)? *(required)*

Select one option.
○ Yes
○ No

54. How often are the dead birds removed from the poultry house? *(required)*

Select one option.
○ Daily
○ Every two days
○ Less frequent than once every two days

55. What is the stocking density (according to final weight) of the poultry house? *(required)*

Select one option.
○ ≤ 33 kg/m²
○ 34 kg/m²
○ 35 kg/m²
○ 36 kg/m²
○ 37 kg/m²
○ 38 kg/m²
○ 39 kg/m²
○ 40 kg/m²
○ 41 kg/m²
○ 42 kg/m²
○ > 42 kg/m²

56. Are there different age categories of poultry present on your farm? *(required)*

Select one option.
○ Yes
○ No

J. Cleaning and disinfection

57. Are vehicle disinfection baths or channels available at the entrance of the farm? *(required)*

Select one option.
○ Yes
○ No (Go to question 59)

58. Are the vehicle disinfection baths/channels always used? *(required)*

Select one option.
○ Yes
○ No

59. Are the poultry houses cleaned after each production cycle? *(required)*

Select one option.
○ Yes
○ No

60. Are the poultry houses disinfected after each production cycle? *(required)*

Select one option.
○ Yes
○ No

61. Is the efficacy of cleaning and disinfection checked after each production cycle (e.g. hygienogram or swabs)? *(required)*

Select one option.
○ Always
○ Sometimes
○ Never

62. Is the loading and unloading area cleaned and disinfected after each production cycle? *(required)*

Select one option.
○ Yes
○ No

63. How long (in days) does the sanitary break after each production cycle last? *(required)*

Select one option.
○ Less than 3 days
○ Between 3 and 8 days
○ More than 8 days

64. Is there a farm hygiene lock available? *(required)*

Select one option.
○ Yes
○ No (Go to question 67)

65. Is there a strict separation between the clean and the dirty area of the farm hygiene lock? *(required)*

Select one option.
○ Yes
○ No

66. Is there a changing room with farm-specific clothes and shoes in the farm hygiene lock? *(required)*

Select one option.
○ Yes
○ No

67. Is there a house hygiene lock present at every poultry house? *(required)*

Select one option.
○ Yes
○ No (Go to question 71)

68. Is there a strict separation between the clean and the dirty area of the house hygiene lock? *(required)*

Select one option.
○ Yes
○ No

69. Is there a disinfection bath/boot washer present in the house hygiene lock? *(required)*

Select one option.
○ Yes
○ No

70. Is it possible to wash and disinfect your hands in the house hygiene lock? *(required)*

Select one option.
○ Yes
○ No

71. Is there a disinfection bath/boot washer at the entrance of the farm? *(required)*

Select one option.
○ Yes
○ No (Go to question 73)

72. Is the fluid of the disinfection baths immediately changed when visually contaminated? *(required)*

Select one option.
○ Yes
○ No

73. Is the drinking water system properly cleaned and disinfected both on the in- and outside after each production cycle? *(required)*

Select one option.
○ Always
○ Sometimes
○ Never

74. Are the feeding systems properly cleaned and disinfected both on the in- and outside after each production cycle? *(required)*

Select one option.
○ Always
○ Sometimes
○ Never

75. Are the feed silos cleaned and disinfected on the inside? *(required)*

Select one option.
○ Yes, after every one or two production cycle(s)
○ Sometimes
○ Never

K. Materials and measures between compartments

76. Is there a protocol for the cleaning and disinfection of material after each production cycle and is this protocol always abided by? *(required)*

Select one option.

○ Yes
○ No

77. Are there multiple poultry houses present on the farm? *(required)*

Select one option.

○ Yes
○ No (Go to end)

78. Has clearly recognisable, separate material been foreseen for each poultry house? *(required)*

Select one option.

○ Yes
○ No

79. Are poultry house-specific clothes and boots available? *(required)*

Select one option.

○ Yes
○ No
