# Supplementary material for: Strengths, challenges, and variations - insights into biosecurity practices in Swedish poultry production following HPAI outbreaks
Source: Poult Sci. 2025 Sep 19;104(12):105871. doi: 10.1016/j.psj.2025.105871 (PMC12529493; doi:10.1016/j.psj.2025.105871)
Supplement: Supplementary file 3 — Supplementary material 3: Biocheck Laying hens_EN [file mmc3.pdf]

FACULTY OF VETERINARY MEDICINE
accredited by EAEVE

biocheck .ugent

Version of Biocheck accessed in December 2021 and used for study: Grant et al., 2025, Strengths, challenges and variations - insights into biosecurity practices in Swedish poultry production following HPAI outbreaks

BIOCHECK POULTRY

Laying hens

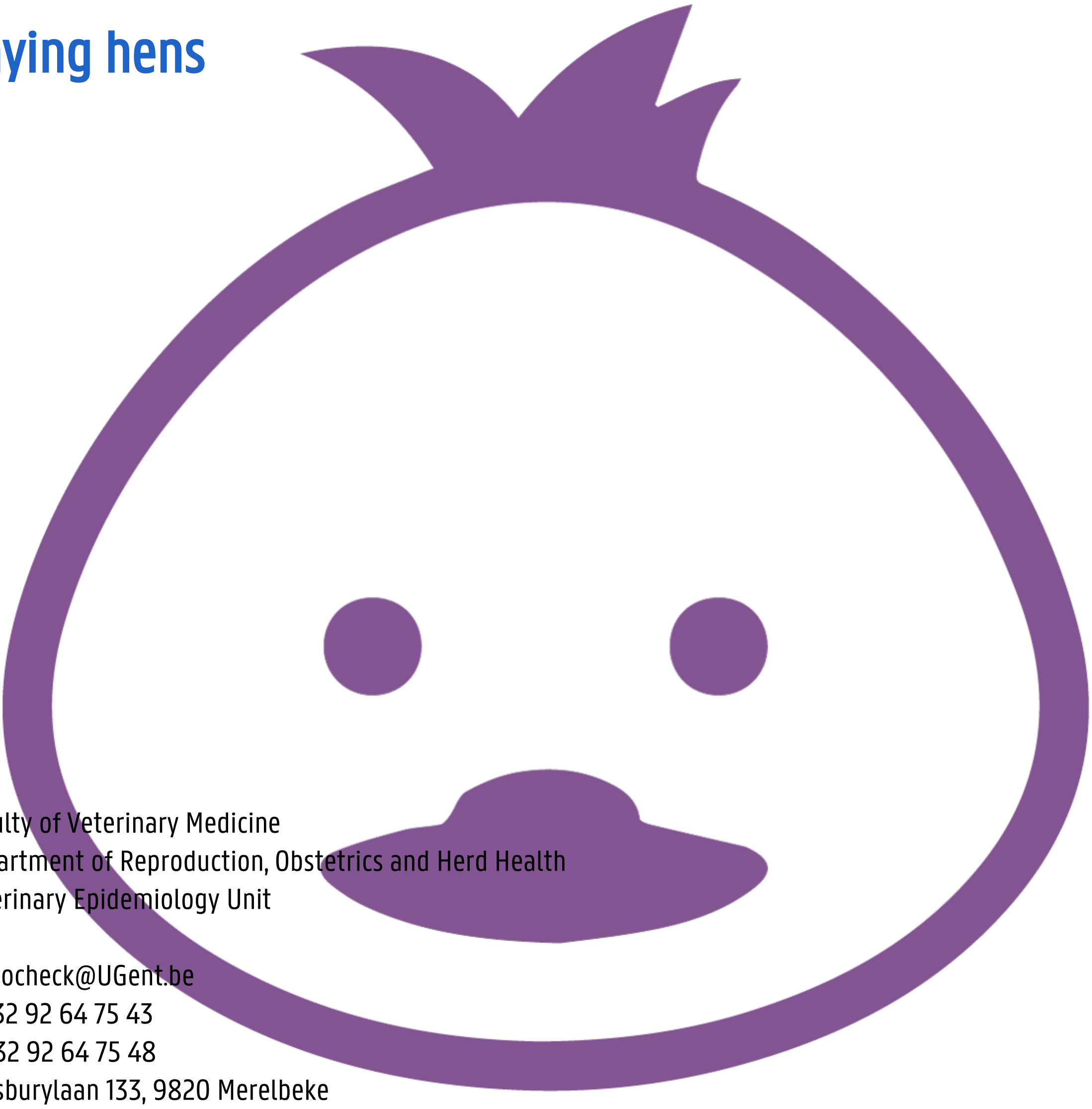

Faculty of Veterinary Medicine
Department of Reproduction, Obstetrics and Herd Health
Veterinary Epidemiology Unit

E: Biocheck@UGent.be
T: +32 92 64 75 43
+32 92 64 75 48
Salisburylaan 133, 9820 Merelbeke
Belgium

www.biocheck.ugent.be

GHENT UNIVERSITY

˜. Farm characteristics

I. How many laying hens are there on the farm?

.... laying hens

II. How many years of experience in keeping poultry does the person in charge have?

.... years

III. How many people are working on the farm?

.... persons

IV. How old (in years) is the oldest building in which poultry is being kept?

.... years

Va. Are there, besides the laying hens, any other farm animals present at your farm?

Select one option.
○ Yes
○ No (Go to question VI)

Vb. What other animal(s) is (are) present at your farm?

Check any that apply.
☐ Pigs
☐ Horses
☐ Cattle
☐ Sheep/goats
☐ Others

VI. How old (in years) is the newest building in which poultry is being kept?

.... years

VII. What type of housing system is used on the farm?

Select one option.
○ Cages
○ Enriched cages
○ Flooring system
○ Aviary
○ Free-range system

A. Purchase of one-day-old chicks

1. Is there a rearing facility present on the farm? *(required)*

Select one option.
○ Yes
○ No (Go to question 5)

2. Are your one-day-old chicks (during the last 2 years) always bought from the same original source? *(required)*

Select one option.
○ Always the same supplier
○ Sometimes a different supplier

3. Are the bought one-day-old chicks first delivered at your farm, i.e. before other farms are supplied by the same transport vehicle? *(required)*

Select one option.
○ Always
○ Sometimes
○ Never

4. Are the transport vehicles (including the transport crates and containers) cleaned and disinfected before the one-day-old chicks are loaded? *(required)*

Select one option.
○ Always
○ Sometimes
○ Never

B. Purchase of laying hens

5. Are your laying hens (during the last 2 years) always bought from the same original source? *(required)*

Select one option.
○ Always the same supplier
○ Sometimes a different supplier

6. Whenever laying hens are bought from another farm, is proof requested to ensure that the sanitary statute and health management of the farm of origin is equal or higher than your own farm? *(required)*

Select one option.
○ Yes
○ No

7. Are the bought laying hens first delivered to your farm, i.e before other farms are supplied by the same transport vehicle? *(required)*

Select one option.
○ Always
○ Sometimes
○ Never

8. Are the transport vehicles (including the transport crates and containers) for poultry cleaned and disinfected before the laying hens are loaded? *(required)*

Select one option.
○ Always
○ Sometimes
○ Never

9. What happens with the laying hens after their first production cycle? *(required)*

Select one option.
○ The laying hens are always slaughtered
○ The laying hens are sometimes kept for a second production period after moulting
○ The laying hens are always kept for a second production period after moulting

C. Depopulation and transport of hens

10. Is the transport vehicle (including the transport crates and containers) for poultry empty on arrival at the farm? *(required)*

Select one option.
○ Always
○ Sometimes (Go to question 13)
○ Never (Go to question 13)

11. Is the transport vehicle (including the transport crates and containers) for poultry free from visible dirt on arrival at the farm? *(required)*

Select one option.
○ Always
○ Sometimes (Go to question 13)
○ Never (Go to question 13)

12. Is the transport vehicle (including the transport crates and containers) for poultry cleaned and disinfected on arrival at the farm? *(required)*

Select one option.
○ Always
○ Sometimes
○ Never

13. Do the driver and the catching team receive and wear farm-specific or disposable clothes during the loading of poultry? *(required)*

Select one option.
○ Always
○ Sometimes
○ Never

14. Do the driver and the catching team receive and wear farm-specific or disposable shoes during the loading of poultry? *(required)*

Select one option.
○ Always
○ Sometimes
○ Never

15. Do the driver and the catching team wash and disinfect their hands before entering the poultry houses? *(required)*

Select one option.
○ Always
○ Sometimes
○ Never

16. Are the transport crates and containers loaded and unloaded from the transport vehicle with farm-specific equipment (e.g. loader)? *(required)*

Select one option.
○ Yes
○ No

17. Are the transport crates and containers transported in- and outside the poultry houses with farm-specific equipment (e.g. loader)? *(required)*

Select one option.
○ Yes
○ No

18. Are the transport vehicles for poultry disinfected when entering the farm (e.g. driving through disinfection baths / spray system / etc..)? *(required)*

Select one option.
○ Always
○ Sometimes
○ Never

19. In how many steps does the depopulation of a poultry house take place? *(required)*

Select one option.
○ In one step
○ In two steps
○ In more than two steps

D. Transport of eggs

20. Are the eggs that are ready for transport stored in a specific storeroom (i.e in a room different from the egg room)? *(required)*

Select one option.

○ Yes
○ No

21. Does the driver have access to the egg facilities of the farm? *(required)*

Select one option.

○ Yes, but only to the egg room
○ Yes, but only to the specific storeroom
○ Yes, the driver has access to both the egg room and specific storeroom
○ No, the driver doesn't have access at all

22. Is the transport vehicle for the eggs empty on arrival at the farm? *(required)*

Select one option.

○ Always
○ Sometimes (Go to question 25)
○ Never (Go to question 25)

23. Is the transport vehicle for the eggs cleaned and disinfected before entering the farm? *(required)*

Select one option.

○ Always
○ Sometimes
○ Never

24. Are the transport vehicles for eggs disinfected when entering the farm (e.g. driving through disinfection baths / spray system / etc..)? *(required)*

Select one option.

○ Yes
○ No

25. Are eggs being sold at the farm? *(required)*

Select one option.

○ Yes
○ No (Go to question 27)

26. Do people enter the farm to buy the eggs? *(required)*

Select one option.

○ Yes, they enter the farm; but they stay outside the egg room or specific storeroom
○ Yes, they enter the farm and they go inside the egg room and/or specific storeroom
○ No, the eggs can be bought without entering the farm (e.g. from the public road)

E. Feed and water

27. Is the farm site divided into a clean and dirty area? *(required)*

The clean road/area is the area of the production site with restricted access, i.e. this is the area where only animals from the farm, persons after they have applied the hygienic measures in the hygiene lock, and farm-specific materials and vehicles are allowed. The dirty area comprises all other parts of the farm where visitors, external vehicles, ... have access to. The dirty area also includes the carcass storage facility.

Select one option.
○ Yes
○ No (Go to question 30)
○ I don't know (Go to question 30)

28. Are the clean and dirty area of the farm site clearly separated? *(required)*

Select one option.
○ Yes
○ No

29. Can the feeding company fill up the silos/deliver feed without entering the clean area? *(required)*

Select one option.
○ Yes
○ Only some of them
○ No

30. Are the transport vehicles for feed always disinfected when entering the farm (e.g. driving through disinfection baths / spray system / etc..)? *(required)*

Select one option.
○ Yes
○ No

31. Does the feed supplier have access to the poultry houses and is direct contact with the poultry possible? *(required)*

Select one option.
○ Always
○ Sometimes
○ Never

32. Are the feed silos or the feed storage rooms (storage of complete feed or concentrate) completely sealed against water, birds and vermin? *(required)*

Select one option.
○ Yes
○ No

33. How often a year does the feeding company fill up the silos or deliver feed? *(required)*

Select one option.
○ Less than 20 times a year
○ Between 20 and 35 times a year
○ More than 35 times a year

34. How often are bacteriological analyses of the drinking water performed? *(required)*

Select one option.
○ At least once a year
○ Every two years
○ Less frequent than every two years
○ Never (Go to question 36)

35. Where is the water sample for the bacteriological analyses taken? *(required)*

Select one option.
○ At the source
○ At the last drinker
○ At both locations, i.e. at the source and at the last drinker

F. Removal of manure and carcasses

36. Is manure being stored on the farm? *(required)*

Select one option.
○ Yes
○ No (Go to question 38)

37. Is the manure stored in a fully closed container or compartment? *(required)*

Select one option.
○ Yes
○ No

38. Is the manure removed and disposed of appropriately through the dirty road? *(required)*

Select one option.
○ Yes
○ No

39. Is there a separate carcass storage? *(required)*

Select one option.
○ Yes
○ No (Go to question 43)
○ No, but there's no need for a separate carcass storage, because the carcasses are immediately processed (Go to question 43)

40. Is the carcass storage space protected from vermin, cats and/or dogs? *(required)*

Select one option.
○ Yes, it's completely protected
○ It's only partially protected
○ No

41. Is this carcass storage space cleaned and disinfected after each collection? *(required)*

Select one option.
○ Always
○ Sometimes
○ Never

42. Is the carcass storage cooled? *(required)*

Select one option.
○ Yes
○ No

43. What happens with the carcasses? *(required)*

Select one option.
○ The carcasses are composted
○ The carcasses are buried/burned (Go to question 45)
○ The carcasses are stored and collected by a rendering company (Go to question 46)

44. Are the carcasses composted in a closed system? *(required)*

Select one option.
○ Yes, they are composted inside a building that can be completely closed
○ Yes, they are composted outside, enclosed with plastic
○ No

Continue to question 47.

45. How are the carcasses buried/burned? *(required)*

Select one option.
○ They are burned in an approved incinerator on the farm (Go to question 47)
○ They are buried in the appropriate soil on the farm (Go to question 47)
○ Other

45.1. Where? *(required)*

………

Continue to question 47.

46. Can the carcasses be collected by the rendering company without entering the farm (e.g. from the public road)? *(required)*

Select one option.
○ Yes
○ No

47. Are carcasses manipulated with gloves, or are hands cleaned and disinfected after manipulation of carcasses? *(required)*

Select one option.
○ Always
○ Sometimes
○ Never

48. Is the material used for the removal of dead birds out of the poultry houses (e.g. buckets, wheelbarrow) cleaned and disinfected after each use? *(required)*

Select one option.

○ Always
○ Sometimes
○ Never

G. Visitors and farmworkers

49. Are visitors obliged to notify you of their presence before entering the poultry houses (e.g. visitor's register)? *(required)*

Select one option.
○ Yes
○ No

50. Is a poultry-free period (longer than 12 hours) expected of all visitors before they are allowed to enter the poultry houses? *(required)*

Select one option.
○ Yes
○ No

51. Is there a farm hygiene lock available and is it always used by visitors when they enter the poultry houses? *(required)*

Select one option.
○ Yes
○ No (Go to question 55)

52. Are all poultry houses only accessible for visitors through the farm hygiene lock? *(required)*

Select one option.
○ Yes
○ No

53. Is there a strict separation between the clean and the dirty area of the farm hygiene lock? *(required)*

Select one option.
○ Yes
○ No

54. Is there a changing room with farm-specific clothes and shoes in the farm hygiene lock? *(required)*

Select one option.
○ Yes
○ No

55. Do visitors and farmworkers have to wear farm-specific clothing before they are allowed to enter the farm? *(required)*

Select one option.
○ Yes
○ No

56. Do visitors and farmworkers have to wear farm-specific shoes/overshoes before they are allowed to enter the farm? *(required)*

Select one option.
○ Yes
○ No

57. Do visitors and farmworkers have to wash and disinfect their hands before they are allowed to enter the farm? *(required)*

Select one option.
○ Yes
○ No

58. How many times per year is access to the poultry houses granted to visitors? *(required)*

Select one option.
○ Access is never granted
○ Access is granted, but less than 12 times a year
○ Access is granted more than 12 times a year

59. Are there any farmworkers who also keep poultry or any other type of bird at home? *(required)*

Select one option.
○ Yes
○ No

60. Are there any farmworkers who also work on other poultry farms? *(required)*

Select one option.
○ Yes
○ No

H. Material supply

61. Is there any material being shared with other farms that enters the poultry houses and/or has contact with your poultry? *(required)*

Select one option.

○ Yes
○ No

62. Are specific measures taken for the introduction of material (e.g. UV-disinfection unit, alcohol disinfection)? *(required)*

Select one option.

○ Yes
○ No

I. Infrastructure and biological vectors

63. Does the poultry have access to the outside, i.e. the open air? *(required)*

Select one option.
○ Yes
○ No (Go to question 65)

64. Is the outdoor area enclosed with nets on all sides (including the ceiling)? *(required)*

Select one option.
○ Yes
○ No

65. Are there bird- and vermin-proof grids placed on the air inlets? *(required)*

Select one option.
○ Yes
○ No

66. Is the farm fenced? *(required)*

Select one option.
○ Yes, it's completely fenced
○ It's only partially fenced
○ No

67. Is the outside of the farm (around the walls) paved and clean (e.g. removal of weeds, waste, ...)? *(required)*

Select one option.
○ Yes, it's completely paved and clean
○ It's only partially paved and clean
○ No

68. Are vermin (i.e. rats, mice, etc.) considered to be a problem at the farm? *(required)*

Select one option.
○ Often
○ Sometimes
○ Never

69. Is a rodent control programme present on the farm? *(required)*

Select one option.
○ Yes, a professional pest control company has been hired
○ Yes, I have established my own pest control programme
○ No

70. Do pets have access to the poultry houses (including the hygiene lock and egg room)? *(required)*

Select one option.

○ Yes
○ No

71. Is “backyard”-poultry or any other type of bird being kept? *(required)*

Select one option.

○ Yes
○ No

72. Are any other farm animals being kept on the same farm site? *(required)*

Select one option.

○ Yes
○ No

J. Location of the farm

73. Is there stagnant or running water within a 1-kilometre radius (0.6 miles) of the farm? *(required)*

Select one option.

○ Yes
○ No

74. At what distance (straight-line) is the nearest neighbouring poultry farm located? *(required)*

Select one option.

○ Less than 500 metres (Less than 0.3 miles)
○ Between 500 metres and 1 kilometre (between 0.3 and 0.6 miles)
○ More than 1 kilometre (more than 0.6 miles)

75. Is manure from other poultry farms spread on the neighbouring farmlands (within a 500-metre (0.3 miles) radius)? *(required)*

Select one option.

○ Often
○ Sometimes
○ Never

76. Does animal transport frequently occur (i.e. minimum once a day) via the public road (road less than 100 metres (328 feet) from your farm) where your farm is located at (e.g. due to the location of a slaughterhouse in the neighbourhood...)? *(required)*

Select one option.

○ Yes
○ No

K. Disease management

77. Is the poultry checked on a daily basis? *(required)*

Select one option.
○ Yes
○ No

78. Is a poultry health management programme, for which regular farm visits (e.g. by your veterinarian(s)) are performed, present on your farm? *(required)*

Select one option.
○ Yes
○ No (Go to question 80)

79. Are autopsies of culled and dead birds systematically performed during these visits? *(required)*

Select one option.
○ Always
○ Sometimes
○ Never

80. How often are the poultry houses checked to remove dead birds? *(required)*

Select one option.
○ Twice or more a day
○ Once a day
○ Less frequent than once a day

81. Are there different age categories of poultry present on your farm? *(required)*

Select one option.
○ Yes, there are different age categories within one poultry house (Go to question 83)
○ Yes, there are different age categories, which are sorted by poultry house
○ No (Go to question 83)

82. Is farm work, per poultry house, performed from young to older birds? *(required)*

Select one option.
○ Yes
○ No

L. Cleaning and disinfection

83. Are the poultry houses cleaned after each production cycle? *(required)*

Select one option.
○ Yes, dry and wet cleaning is performed after each production cycle
○ Yes, however, only dry cleaning is performed after each production cycle (Go to question 86)
○ No (Go to question 86)

84. Are the poultry houses soaked with water before the start of cleaning? *(required)*

Select one option.
○ Always
○ Sometimes
○ Never

85. Is detergent added to the water during cleaning? *(required)*

Select one option.
○ Always
○ Sometimes
○ Never

86. Are the poultry houses disinfected after each production cycle? *(required)*

Select one option.
○ Always
○ Sometimes
○ Never (Go to question 88)

87. Are the poultry houses dry before the start of the disinfection? *(required)*

Select one option.
○ Always
○ Sometimes
○ Never

88. Is the efficacy of cleaning and disinfection checked, with for example a hygienogram, swabs, ..., after each production cycle? *(required)*

Select one option.
○ Always
○ Sometimes
○ Never

89. Are the central corridor and other common places cleaned after each production cycle? *(required)*

Select one option.
○ Yes, dry and wet cleaning is performed after each production cycle
○ Yes, however, only dry cleaning is performed after each production cycle (Go to question 91)
○ No (Go to question 91)

90. Are the central corridor and other common places disinfected after each production cycle? *(required)*

Select one option.
○ Yes
○ No

91. Is the egg room regularly cleaned? *(required)*

Select one option.
○ Yes, after each outgoing egg transport
○ Yes, after each production cycle
○ No (Go to question 93)

92. Is the egg room regularly disinfected? *(required)*

Select one option.
○ Yes, after each outgoing egg transport
○ Yes, after each production cycle
○ No

93. Is the material/machinery used for egg collecting regularly cleaned? *(required)*

Select one option.
○ Yes, after each outgoing egg transport
○ Yes, after each production cycle
○ No (Go to question 95)

94. Is the material/machinery used for egg collecting regularly disinfected? *(required)*

Select one option.
○ Yes, after each outgoing egg transport
○ Yes, after each production cycle
○ No

95. Is the loading and unloading area cleaned and disinfected after each production cycle? *(required)*

Select one option.
○ Yes
○ No

96. Is the drinking water system properly cleaned and disinfected both on the in- and outside after each production cycle? *(required)*

Select one option.
○ Always
○ Sometimes
○ Never

97. Are the feeding systems (e.g. storage bins, augers, hoppers and chain feeders) cleaned and disinfected after each production cycle? *(required)*

Select one option.
○ Always
○ Sometimes
○ Never

98. Are the feed silos cleaned and disinfected on the inside? *(required)*

Select one option.
○ Yes, after every one or two production cycle(s)
○ Sometimes
○ Never
○ Silo's aren't used

M. Materials and measures between compartments

99. Are there multiple poultry houses present on the farm? *(required)*

Select one option.
○ Yes
○ No (Go to question 110)

100. Is there a house hygiene lock present at every poultry house? *(required)*

Select one option.
○ Yes
○ No (Go to question 103)

101. Is there a strict separation between the clean and the dirty area of the house hygiene lock? *(required)*

Select one option.
○ Yes
○ No

102. Is there a changing room with poultry house-specific clothing and shoes in the house hygiene lock? *(required)*

Select one option.
○ Yes
○ No

103. Do visitors and farmworkers have to wear poultry house-specific clothes before they are allowed to enter the poultry house? *(required)*

Select one option.
○ Yes
○ No

104. Do visitors and farmworkers have to wear poultry house-specific shoes/overshoes before they are allowed to enter the poultry house? *(required)*

Select one option.
○ Yes
○ No

105. Do visitors and farmworkers have to wash and disinfect their hands before they are allowed to enter the poultry house? *(required)*

Select one option.
○ Yes
○ No

106. Is there a disinfection bath/boot washer at the entrance of each poultry house? *(required)*

Select one option.
○ Yes
○ No

107. Is the fluid of the disinfection baths/boot washers immediately changed when visually contaminated? *(required)*

Select one option.
○ Yes
○ No

108. Has clearly recognisable, separate material been foreseen for each poultry house? *(required)*

Select one option.
○ Yes
○ No

109. Is there a protocol for the cleaning and disinfection of material after each production cyle and is this protocol abided by? *(required)*

Select one option.
○ Yes
○ No

N. Egg management

110. Are the eggs collected in the poultry house itself, or does the collection take place in a separate room close to the poultry house (e.g. in connection with an automatic belt)? *(required)*

Select one option.
○ In the poultry house itself
○ In a separate room next door

111. Do you have a fully automatic egg collection system or are people manually doing the collection and/or transportation of the eggs to the egg room? *(required)*

Select one option.
○ There is a fully automatic system (Skip question 113)
○ Manual collection and/or transportation of the eggs by farmworkers

112. Are the farmworkers active in the poultry houses, also working in the egg room? *(required)*

Select one option.
○ Yes
○ No

113. Do the farmworkers, who collect and/or transport the eggs to the sorting/packaging room, take strict preventive measures between the egg sorting/packaging room and the poultry houses? *(required)*

Select one option.
○ Always
○ Sometimes
○ Never

114. Are dirty, cracked and/or broken eggs removed from the collection systems as early as possible and handled separately? *(required)*

Select one option.
○ Yes
○ No

115. Are the egg belts, belt brushes and other egg handling equipment cleaned after each production cycle? *(required)*

Select one option.
○ Always
○ Sometimes
○ Never (Go to question 117)

116. Are the egg belts, belt brushes and other egg handling equipment disinfected after each production cycle? *(required)*

Select one option.
○ Always
○ Sometimes
○ Never

117. Are disposable egg trays being used to transport the eggs? *(required)*

Select one option.
○ Yes (Go to end)
○ No

118. What are the egg trays made of? *(required)*

Select one option.
○ Plastic
○ Cardboard

119. Are the egg trays regularly cleaned? *(required)*

Select one option.
○ Yes, after each egg transportation
○ Yes, after each production cycle
○ No (Go to end)

120. Are the egg trays regularly disinfected? *(required)*

Select one option.
○ Yes, after each egg transportation
○ Yes, after each production cycle
○ No
